# TDP-43 stabilises the processing intermediates of mitochondrial transcripts

**DOI:** 10.1038/s41598-017-06953-y

**Published:** 2017-08-09

**Authors:** Keiichi Izumikawa, Yuko Nobe, Harunori Yoshikawa, Hideaki Ishikawa, Yutaka Miura, Hiroshi Nakayama, Takashi Nonaka, Masato Hasegawa, Naohiro Egawa, Haruhisa Inoue, Kouki Nishikawa, Koji Yamano, Richard J. Simpson, Masato Taoka, Yoshio Yamauchi, Toshiaki Isobe, Nobuhiro Takahashi

**Affiliations:** 1 Global Innovation Research Organizations, Tokyo University of Agriculture and Technology, 3-5-8 Saiwai-cho, Fuchu, Tokyo 183-8509 Japan; 2 Core Research for Evolutional Science and Technology (CREST), Japan Science and Technology Agency (JST), Sanbancho 5, Chiyoda-ku, Tokyo 102-0075 Japan; 3 Department of Chemistry, Graduate School of Science, Tokyo Metropolitan University, 1-1 Minami-ohsawa, Hachioji, Tokyo 192-0397 Japan; 4 Centre for Gene Regulation & Expression, School of Life Sciences, University of Dundee, Dow Street, Dundee, DD1 5EH UK; 5 Biomolecular Characterization Unit, RIKEN Center for Sustainable Resource Science, 2-1 Hirosawa, Wako, Saitama 351-0198 Japan; 6 Department of Dementia and Higher Brain Function/Dementia Research Project, Tokyo Metropolitan Institute of Medical Science, 2-1-6 Kamikitazawa, Setagaya-ku, Tokyo 156-8506 Japan; 7 Center for iPS Cell Research and Application (CiRA), Kyoto University, Kyoto, 606-8507 Japan; 8 Cellular and Structural Physiology Institute (CeSPI), Nagoya University, Nagoya, 464-8601 Japan; 9 Ubiquitin project, Tokyo Metropolitan Institute of Medical Sciences, Setagaya-ku, Tokyo 156-8506 Japan; 10 La Trobe Institute for Molecular Science (LIMS), LIMS Building 1, Room 412 La Trobe University, Bundoora, Victoria 3086 Australia

## Abstract

The 43-kDa trans-activating response region DNA-binding protein 43 (TDP-43) is a product of a causative gene for amyotrophic lateral sclerosis (ALS). Despite of accumulating evidence that mitochondrial dysfunction underlies the pathogenesis of TDP-43–related ALS, the roles of wild-type TDP-43 in mitochondria are unknown. Here, we show that the small TDP-43 population present in mitochondria binds directly to a subset of mitochondrial tRNAs and precursor RNA encoded in L-strand mtDNA. Upregulated expression of TDP-43 stabilised the processing intermediates of mitochondrial polycistronic transcripts and their products including the components of electron transport and 16S mt-rRNA, similar to the phenotype observed in cells deficient for mitochondrial RNase P. Conversely, TDP-43 deficiency reduced the population of processing intermediates and impaired mitochondrial function. We propose that TDP-43 has a novel role in maintaining mitochondrial homeostasis by regulating the processing of mitochondrial transcripts.

## Introduction

The trans-activating response region DNA-binding protein 43 (TDP-43) is a member of the family of heterogeneous nuclear ribonucleoproteins and contains two highly conserved RNA recognition motifs (RRMs) and a non-conserved C-terminal region that mediates protein-protein interactions^1^. TDP-43 binds tightly the (UG)_n_ motif^1–4^ and helps regulate several aspects of mRNA biogenesis including transcription, pre-mRNA splicing and export, and mRNA stability^2, 4, 5^. TDP-43 is one of the causative gene products of amyotrophic lateral sclerosis (ALS) that is an adult-onset neurodegenerative disorder characterised by progressive degeneration of upper and lower motor neurons^6, 7.^ The majority of TDP-43 mutations that cause ALS are found in the C-terminal region, although some are found in the RRMs^8–10^. ALS mutant TDP-43 proteins, including those with mutation of the RRMs have an increased half-life in cell models^9, 11, 12^.

Although TDP-43 shuttles between the nucleus and the cytoplasm, it mainly resides in the nucleus under physiological conditions^13^. TDP-43 is cleared from the nuclear compartment and is incorporated into cytoplasmic ubiquitinated and/or hyperphosphorylated inclusions in patients with familial or sporadic ALS and in patients with frontotemporal lobar degeneration^6, 14–16^. Several animal models over-expressing wild type TDP-43 leads to neurodegeneration similar to that observed in ALS^17–24^, accumulate mitochondria in cytoplasmic inclusions in motor neurons^23^, lack mitochondria in motor axon terminals^24^, or have abnormal juxtanuclear aggregates of mitochondria in spinal motor neurons^22^, suggesting that wild type TDP-43 affects mitochondrial function. Supporting this, TDP-43 localises to mitochondria of NSC34 cells, a hybrid cell line that has several motor neuron features^25^. TDP-43–induced cytotoxicity also strongly correlates with mitochondrial functions, including respiratory capacity and respiratory chain activity, in yeast cells expressing human TDP-43^26^. In addition, TDP-43 co-localises with mitochondria in motor neurons, and ALS-associated mutants enhance its localisation in mitochondria^27, 28^. The enhanced localisation of mutant TDP-43 to mitochondria also disrupts mitochondrial dynamics and function^27, 28^. The mitochondrial fusion protein mitofusin 2 (Mfn2) has also been demonstrated to be involved in mitochondrial impairment, but the functional relationship between TDP-43 and Mfn2 is unknown^27^. In this regard, TDP-43 mutants also disrupt the vesicle-associated membrane protein-associated protein B (VAPB)– protein tyrosine phosphatase-interacting protein-51 (PTPIP51) interaction and cellular Ca^2+^ homeostasis, which disrupts mitochondria–endoplasmic reticulum interactions that are implicated in several physiological processes including ATP production, mitochondrial biogenesis, and apoptosis^29^; however, TDP-43 is involved only indirectly in these processes through its ability to activate glycogen synthase kinase-3β^29^. In addition, it was recently proposed that TDP-43 and its mutants impair the assembly of electron transfer complex I by binding to mitochondrial mRNAs encoding NADH-ubiquinone oxidoreductase chain 3 (ND3) and ND6 and inhibiting their translation^28^. Despite the obvious involvement of TDP-43 in mitochondrial function, the direct mitochondrial target of TDP-43 remains elusive.

## Results

### TDP-43 binds mitochondrial (mt-) tRNA^Asn^, mt-tRNA^Gln^ and mt-tRNA^Pro^ directly *in vivo*

**Figure 1:**
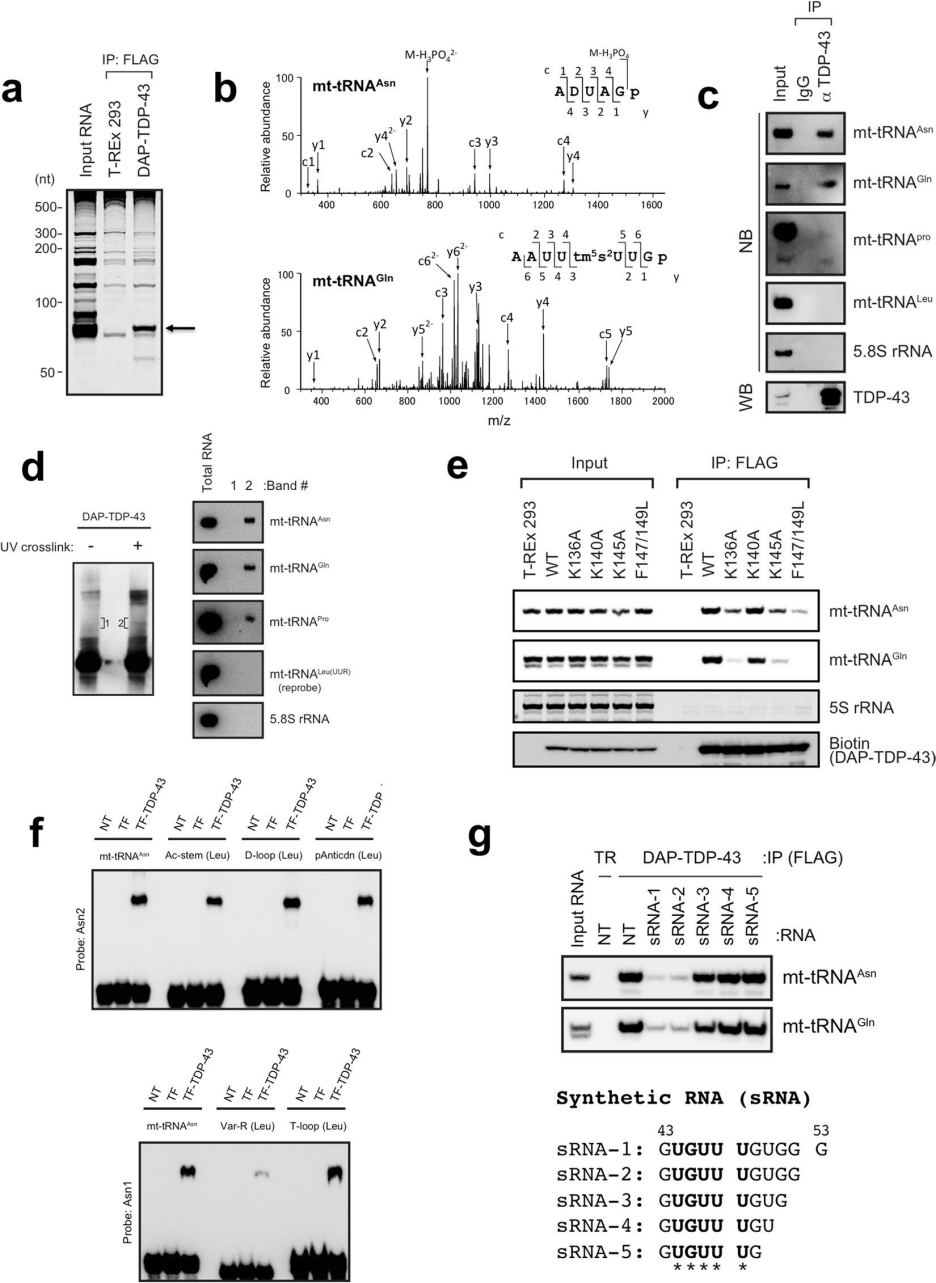
TDP-43 associates with a subset of L-strand-encoded mt-tRNAs. (**a**) RNAs immunoprecipitated (IP) from DAP (double affinity purification tag, see Supplementary Fig. 1a)-TDP-43–expressing cells or T-REx 293 cells were stained with SYBR Gold. (**b**) A typical MS/MS spectrum of the oligonucleotide [ADUAGp]^2−^ that originated from the RNase T1 digest of mt-tRNA^Asn^ (top) or [AAUUtm^5^s^2^UUGp]^2−^ from that of mt-tRNA^Gln^ (bottom). m/z, mass-to-charge ratio. (**c**) Endogenous TDP-43 was immunoprecipitated with anti-TDP-43 (αTDP-43) and analysed by western blotting (WB) with anti-TDP-43. The immunoprecipitated RNAs were detected by northern blotting (NB) with the probes indicated on the right. Rabbit IgG served as a negative control. (**d**) DAP-TDP-43 complex was immunoprecipitated from DAP-TDP-43-expressing T-REx 293 cells with (+) or without (−) UV crosslinking, separated by SDS-PAGE, and detected by WB with horseradish peroxidase (HRP)-conjugated streptavidin (left). The SDS-PAGE gel bands containing RNA-bound DAP-TDP-43 corresponding to the area indicated as 1 and 2 were excised, and RNAs were extracted from the bands and analysed by NB with RNA-specific probes (right). (**e**) Each DAP-TDP-43 mutant bearing a mutation(s) at residue(s) K136, K140, K145, or F147/L149 was immunoprecipitated with anti-FLAG and analysed by WB with HRP-conjugated streptavidin or by NB with the probes indicated on the right. 5S rRNA served as a loading control for RNA and was detected using SYBR Gold staining. (**f**) Recombinant trigger factor–fused TDP-43 (TF-TDP-43) was mixed with synthesised mt-tRNA^Asn^ or its mutant [Ac-stem(Leu), D-loop(Leu), pAntiCdn(Leu), Var-R(Leu) or T-loop(Leu)] shown in Supplementary Fig. 1f, and analysed by electrophoretic mobility shift assay. tRNAs were detected by NB with probes indicated on the left. (**g**) Top: RNAs were immunoprecipitated from TDP-43–expressing T-REx 293 cells in the absence (NT) or presence of synthetic RNA (sRNA-1, sRNA-2, sRNA-3, sRNA-4, or sRNA-5) and analysed by NB with probes indicated on the right. T-REx 293 cells (TR) served as a negative control. Bottom: Sequences of the synthetic RNAs. Asterisks correspond to the sequence that was replaced in Var-R(Leu).

Given that TDP-43 contains RNA recognition motifs (RRMs), we first investigated the association of RNA with exogenously expressed TDP-43 using mass spectrometry (MS) and the Ariadne search engine^30–32^. We established human Flp-In^TM^ T-REx^TM^ 293 (T-REx 293) cell line expressing doxycycline-inducible wild-type human TDP-43 carrying a triple affinity-purification tag (DAP-tag: 6 × histidine, biotin, and FLAG; Supplementary Fig. 1a), and showed that the DAP-TDP-43 expression reduced the level of endogenous TDP-43, suggesting that the exogenous DAP-TDP-43 is behaved as native TDP-43 by constitutive negative feedback mechanism (Supplementary Fig. 1b)^4^. We pulled down TDP-43–associated RNAs and detected ~70-nt RNAs by denaturing urea-PAGE (Fig. 1a). The MS-based identification method revealed that the mitochondrial (mt) genome regions encoding mt-tRNA^Asn^, mt-tRNA^Gln^, and mt-tRNA^Pro^ matched very well with our RNase T1–digested oligonucleotide fragments (Fig. 1b and Supplementary Table 1). These three mt-tRNAs were also detected by northern blotting (Supplementary Fig. 1c) and are encoded in the L-strand of mtDNA. We confirmed the association of endogenous TDP-43 with these mt-tRNAs using anti-TDP-43 immunoprecipitation (Fig. 1c), and the interactions were determined to occur directly within intact cells as assessed with UV cross-linking *in vivo* and immunoprecipitation (Fig. 1d). To determine the region of TDP-43 responsible for the binding with the mt-tRNAs, we established T-REx 293 cell lines expressing TDP-43 mutants that had a domain defect (ΔRRM1, ΔRRM2, ΔGR, Δ315), a point mutation at amino acid residue 136, 140 or 145, or double mutations at residues 147 and 149 (Supplementary Fig. 1d), and showed that residues K136, K145, and F147/149 in the TDP-43 RRM1 are critical for the interaction of TDP-43 with mt-tRNA^Asn^ and mt-tRNA^Gln^ (Supplementary Fig. 1e and Fig. 1e). We also utilised recombinant TDP-43 and synthesised five mt-tRNA^Asn^ mutants in which nucleotide (nt) sequence replacements were made corresponding to a specific region of mt-tRNA^Leu^ [Ac-stem(Leu), D-loop(Leu), pAntiCdn(Leu), Var-R(Leu), or T-loop(Leu)] as well as mt-tRNA^Leu^ as a negative control (Supplementary Fig. 1f). An electrophoretic mobility shift assay revealed that recombinant TDP-43 binds mt-tRNA^Asn^ but not mt-tRNA^Leu^ (Supplementary Fig. 1g) and that the region corresponding to UGUUU (nt 44–48) of mt-tRNA^Asn^ is required for interaction with TDP-43 (Supplementary Fig. 1f and Fig. 1f). Competitive interaction analysis using synthetic RNAs (sRNA-1, sRNA-2, sRNA-3, sRNA-4, and sRNA-5, Fig. 1g) suggested that an additional sequence GUGG (nt 49–53) in mt-tRNA^Asn^ is required for inhibiting the binding to TDP-43 (Fig. 1g).

### TDP-43 is localised in mitochondria in T-REx 293 cells

**Figure 2:**
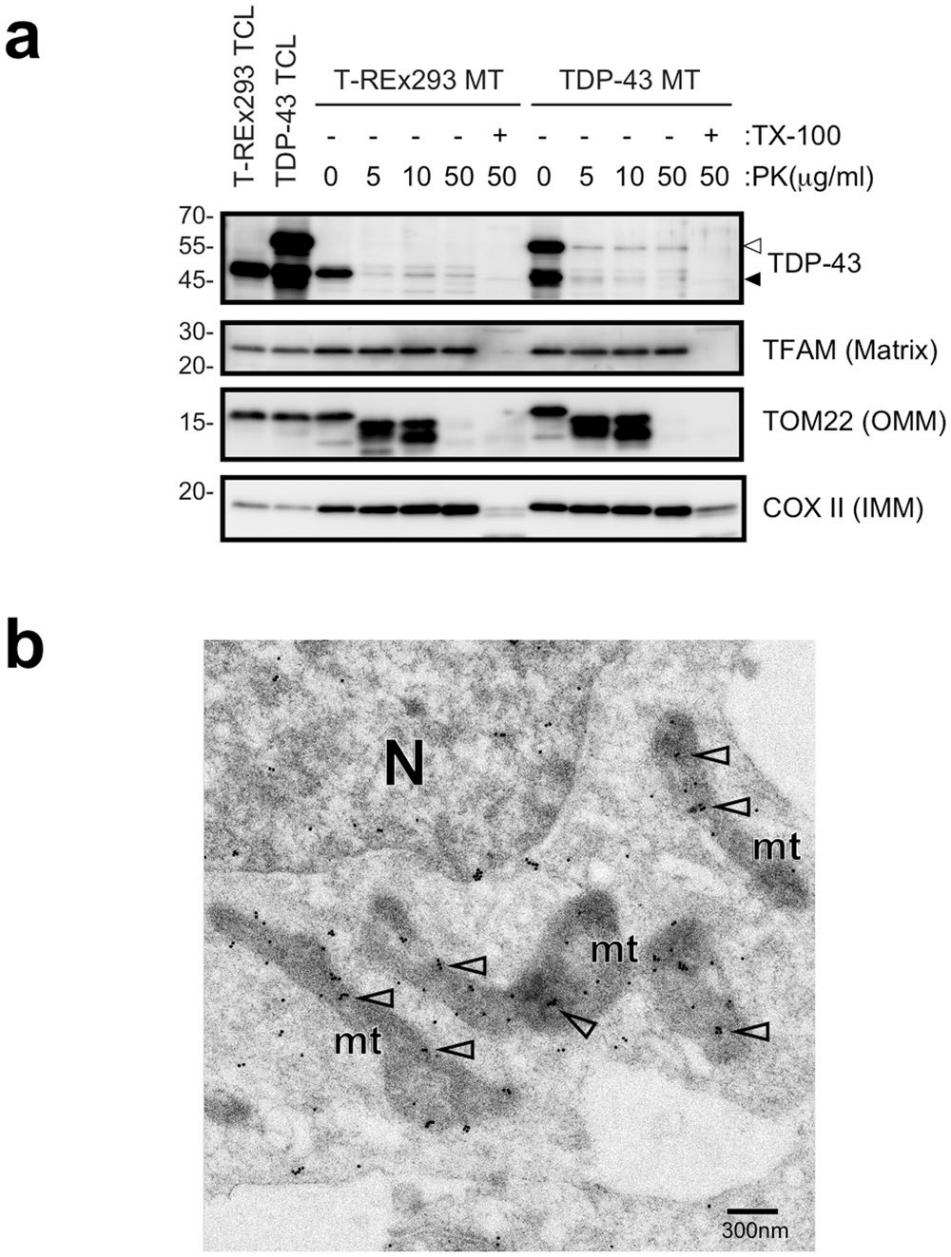
TDP-43 is localised in mitochondria in T-REx 293 cells. (**a**) Mitochondria prepared from T-REx 293 cells (T-REx 293 MT) or DAP-TDP-43 expressing T-REx 293 cells (TDP-43 MT) were treated with proteinase K (PK) in the presence (+) or absence (−) of Triton X-100 (TX-100). Proteins present in the outer mitochondrial membrane (OMM), inner membrane (IMM), or matrix were detected by WB with antibodies indicated on the right. Open arrowhead and closed arrowhead indicate DAP-TDP-43 and endogenous TDP-43, respectively. TCL; Total cell lysate. (**b**) Endogenous TDP-43 was detected within mitochondria of T-REx 293 cells by immunoelectron microscopy using anti-TDP-43 (60019-2-Ig). Open arrowheads indicate stained TDP-43. N, nucleus; mt, mitochondria.

To assess localisation of TDP-43 in mitochondria further, we treated a mitochondrial fraction with proteinase K and detected both endogenous and exogenous TDP-43; TDP-43 was not detected, however, if the mitochondrial membrane was permeabilised with Triton X-100 prior to proteinase K treatment (Fig. 2a). Furthermore, we detected endogenous TDP-43 in mitochondria by immunoelectron microscopy (Fig. 2b). Taken together, these data indicated that TDP-43 has the ability to associate directly with mt-tRNA^Asn^, mt-tRNA^Gln^, and mt-tRNA^Pro^ present within the mitochondria of T-REx 293 cells.

### TDP-43 stabilises not only the cellular levels of the TDP-43-bound mt-tRNAs but also that of TDP-43-unbound mt-tRNA

**Figure 3:**
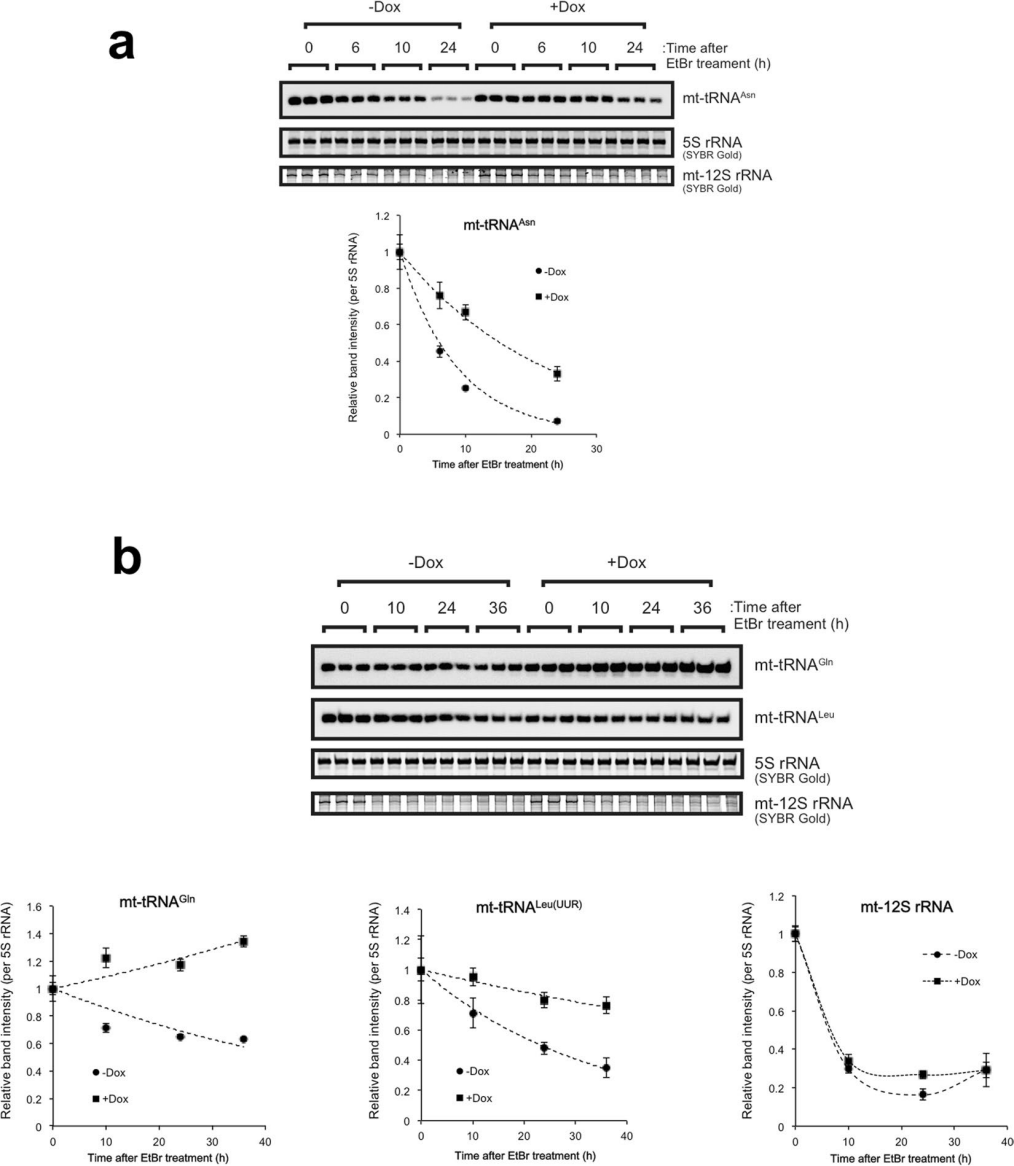
TDP-43 overexpressed increases stabilities of mt-tRNA^Asn^, mt-tRNA^Gln^, and mt-tRNA^Leu(UUR)^. DAP-TDP-43–inducible T-REx 293 cells were treated with or without doxycycline (Dox) for 24 h and then treated with EtBr for the indicated periods. Cells were collected at 0, 6, 10, and 24 h after addition of EtBr for analysis of mt-tRNA^As^ ^n^ by northern blotting (**a**) and at 0, 10, 24, 36 h for analysis of mt-tRNA^Gln^ or mt-tRNA^Leu(UUR)^ (**b**). mt-12S rRNA was detected by SYBR Gold staining and MS-based analysis (**b**). The graphs present data for the average ± SD (three independent experiments) of staining intensities at each time point relative to 0 h. Each band intensity was normalised to that of 5S rRNA detected using SYBR Gold staining.

In human mitochondria, large polycistronic RNA transcripts are generated from the L- and H-strand promoters of mtDNA^33^. The L-strand encodes one oxidative phosphorylation system subunit (ND6) and eight mt-tRNAs (for Pro, Glu, Ser (UCN), Tyr, Cys, Asn, Ala, and Gln) (Supplementary Fig. 2a). Of these, the mt-tRNAs for Asn, Gln, and Pro bound directly to TDP-43. To gain insight into the physiological roles of the binding of TDP-43 to the L-strand-encoded mt-tRNAs, we examined whether TDP-43 is involved in stabilisation of those three mt-tRNAs *via* direct binding. We used ethidium bromide (EtBr) to inhibit mtDNA transcription and first examined the effect of EtBr on stability of L-strand coded mt-RNAs in T-REx 293 cells. EtBr reduced the levels of all of the L-strand-encoded mt-tRNAs with time but it did not affect a genomic tRNA^Met^ (Supplementary Fig. 2b). It also reduced the levels of H-strand-encoded mt-tRNA^Leu(UUR)^ (Supplementary Fig. 2b) and mRNAs for ND1, ND2 and COXI, whereas it did not affect that of 28S rRNA (Supplementary Fig. 2c). We then examined the stabilities of TDP-43–bound mt-tRNA^Asn^ and mt-tRNA^Gln^ and TDP-43–unbound mt-tRNA^Leu(UUR)^ and mt-12S rRNA by inhibiting mtDNA transcription with EtBr before and after doxycycline induction of TDP-43. Overexpression of TDP-43 slowed the degradation of mt-tRNA^Asn^ and mt-tRNA^Gln^ (Fig. 3a and b). Moreover, TDP-43 overexpression unexpectedly slowed the degradation of mt-tRNA^Leu(UUR)^, whereas it had only a negligible effect on the degradation of mt-12S rRNA (Fig. 3b). These results suggested that TDP-43 could increase the abundance of particular mt-tRNAs independent of mtDNA transcription. Although the stabilisation of mt-tRNA^Asn^ and mt-tRNA^Gln^ may be attributable, at least in part, to the direct binding of TDP-43, the stabilisation of mt-tRNA^Leu(UUR)^ upon overexpression of TDP-43 cannot be attributed to stabilisation mediated by direct binding of TDP-43.

### TDP-43 stabilises the L-strand-encoded mt-tRNAs and their precursors

**Figure 4:**
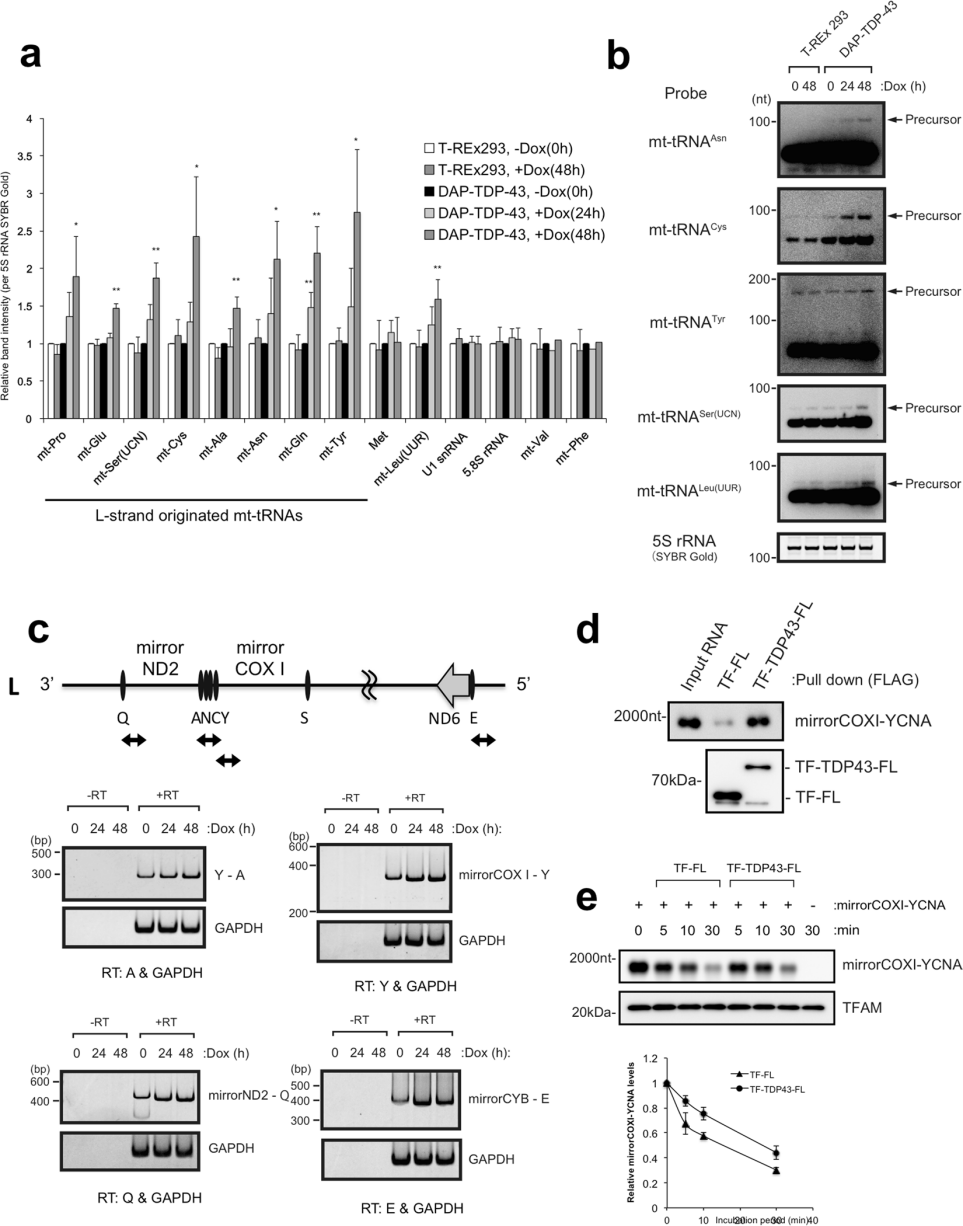
TDP-43 overexpression increases polycistronic transcripts of L- and H-strand mtDNA. (**a**) Relative band intensities of mt-tRNAs in total RNA extracted from DAP-TDP-43–expressing T-REx 293 cells (harvested at 0, 24, and 48 h after induction with doxycycline, Dox) were quantified by northern blotting after polyacrylamide gel electrophoresis. mt-AAA represents mt-tRNA^AAA^; e.g., mt-Pro, mt-tRNA^Pro^, etc. 5S rRNA stained with SYBR Gold served as the loading control. Data are mean ± SD, n = 3–6. **P < 0.01; *P < 0.05 (paired t test). (**b**) Overexposed views of representative northern blots from (**a**) showing precursor bands of each mt-tRNA. 5S rRNA stained with SYBR Gold served as the loading control. (**c**) Total RNA extracted from DAP-TDP-43–expressing T-REx 293 cells harvested at 0, 24, or 48 h after Dox induction were reverse transcribed (+RT) or not reverse transcribed (−RT) with the indicated gene-specific primers for L-strand-specific RT-PCR analysis. The PCR products were separated by PAGE and detected with SYBR Gold staining. The primers amplified the following: a 140-nt region in *GAPDH* mRNA and a 300-nt region of the L-strand transcript containing mt-tRNA^Tyr^, mt-tRNA^Cys^, mt-tRNA^Asn^, and mt-tRNA^Ala^ (Y - A, top left), a 400-nt region between mirrorND2 and mt-tRNA^Gln^ (Q) of the L-strand transcript (bottom left), a 300-nt region between mirrorCOXI and mt-tRNA^Tyr^ (Y) of the L-strand transcript (top right), and a 400-nt region between mirrorCYB and mt-tRNA^Glu^ (E) of the L-strand transcript (bottom right). Schematic diagrams of the corresponding PCR fragments are shown (upper diagram). (**d**) Recombinant TDP-43 fused with trigger factor (TF) and FLAG tag (FL) (TF-TDP43-FL) was mixed with synthesised mirrorCOXI-YCNA (mt-tRNA^Tyr^-mt-tRNA^Cys^-mt-tRNA^Asn^ -mt-tRNA^Ala^; 2000 nt) RNA and pulled down with anti-FLAG beads. mirrorCOXI-YCNA was detected by northern blotting, and TF-FL or TF-TDP43-FL by western blotting with anti-FLAG. (**e**) mirrorCOXI-YCNA was incubated with mitochondrial extract from 293 T cells for the indicated times in the presence of TF-FL or TF-TDP43-FL. TFAM (transcription factor A mitochondrial protein) served as a loading control. Data in the graph represent the mean ± SEM of three independent experiments.

We next used northern blotting to address whether overexpression of TDP-43 affects cellular levels of L-strand-encoded mt-tRNAs first other than those bound by TDP-43, using nuclear genome–encoded RNAs (tRNA^Met^, U1 snRNA, and 5.8S rRNA) as the references. Overexpression of TDP-43 significantly increased the cellular levels of all L-strand-encoded mt-tRNAs (Fig. 4a). Consistent with the result described above (Fig. 3b), it also increased the cellular level of H-strand-encoded mt-tRNA^Leu(UUR)^ (Fig. 4a). In addition, the RNA species detected in the northern blots for mt-tRNA^Asn^, mt-tRNA^Cys^, mt-tRNA^Tyr^, mt-tRNA^Ser(UCN)^, and mt-tRNA^Leu(UUR)^ appeared slightly larger than the mature transcripts upon overexpression of TDP-43 (Fig. 4b). These data suggested that TDP-43 might stabilise the production of L-strand-encoded mt-tRNAs and possibly H-strand-encoded mt-tRNAs at the level of their precursors or polycistronic RNA transcripts.

To explore this possibility, we next investigated the accumulation of L-strand-encoded polycistronic transcripts in TDP-43–expressing T-REx 293 cells by L-strand-specific reverse-transcription PCR (RT-PCR). The RT-PCR primers were designed to amplify the boundaries among mt-tRNAs or between mt-tRNAs and mt-mRNAs in the region containing mt-tRNA^Tyr^ (Y)-mt-tRNA^Ala^ (A), the region complementary to *COXI* mRNA (mirrorCOXI)-Y, mirrorND2-mt-tRNA^Gln^ (Q), and mirrorCYB-mt-tRNA^Glu^ (E) to test for cleavage within these boundary regions. All boundaries were found to increase in abundance with time after doxycycline induction compared with *GAPDH* mRNA (Fig. 4c). In addition, we used a synthetic precursor RNA encompassing a region containing mirrorCOXI and mt-tRNA^Tyr^-mt-tRNA^Cys^-mt-tRNA^Asn^-mt-tRNA^Ala^ (YCNA) and confirmed the direct binding of the precursor RNA to recombinant TDP-43 (Fig. 4d) and the stabilisation of the precursor RNA by recombinant TDP-43 in mitochondrial extracts (Fig. 4e). These results suggested that increased expression of TDP-43 stabilises the mt-tRNA-containing regions of mitochondrial polycistronic transcripts and increases the abundance of their processed products. Thus, TDP-43 appears to participate in the stabilisation of large polycistronic transcripts from L-strand mtDNA and regulates the cellular levels of the precursor and processed mt-tRNA products.

### Overexpression of TDP-43 increases un-processed precursors that are accumulated by the deficiency of mitochondrial RNase P

**Figure 5:**
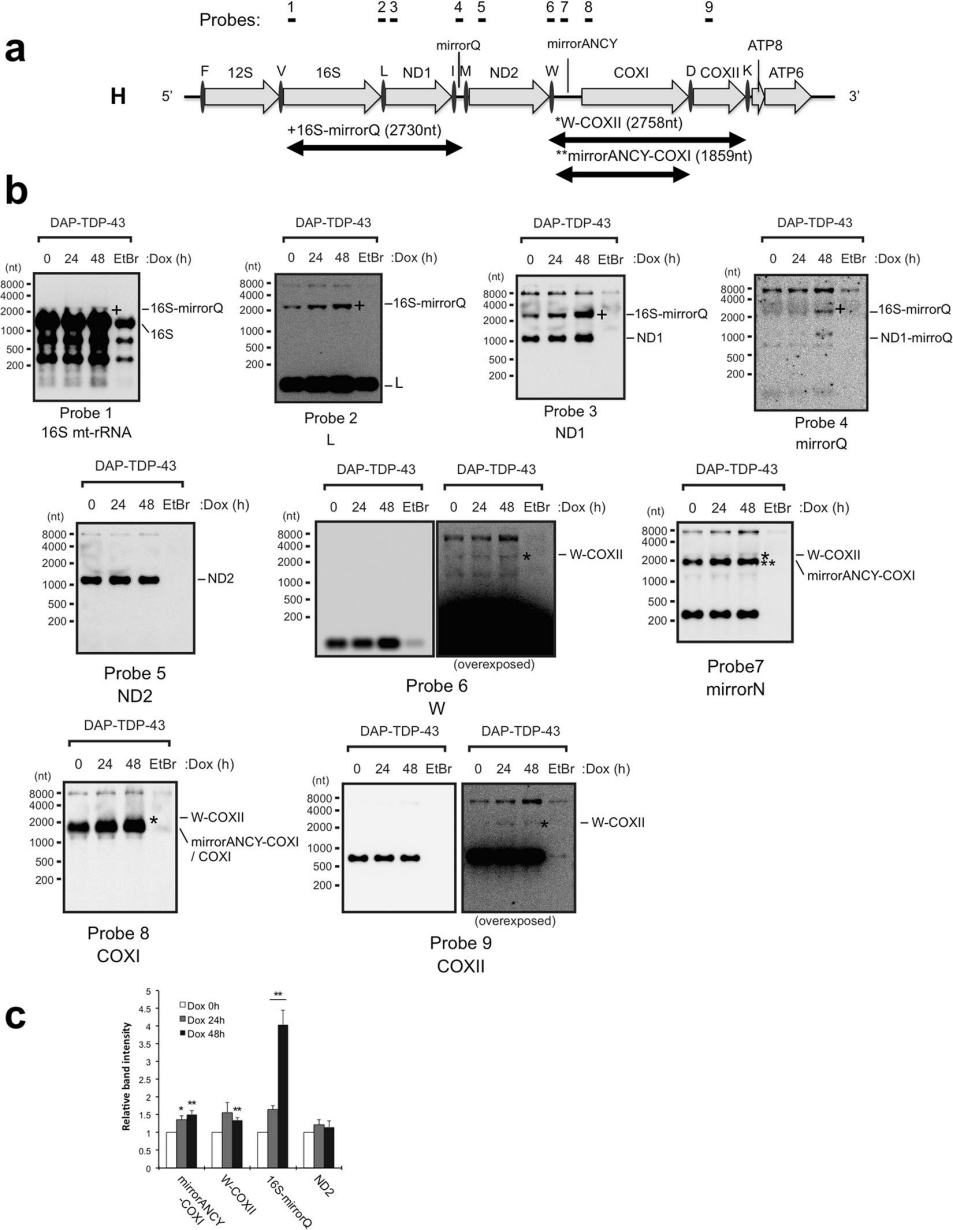
TDP-43 overexpression causes accumulation of intermediates of mitochondrial transcript. (**a**) Schematic diagram of H-strand mitochondrial DNA from mt-tRNA^Phe^ (F) to ATP6, and the corresponding mt-transcripts are shown. Nine probes used for northern blotting are indicated above the diagram. (**b**) Total RNA extracted from DAP-TDP-43-expressing T-REx 293 cells harvested at 0, 24, or 48 h after doxycycline (Dox) induction were analysed by northern blotting with the probes indicated under the figures. EtBr served as a negative control for the mt-transcripts. The processing intermediates of mt-transcripts identified by northern blotting are indicated under the diagram and to the right of each panel. (**c**), Graphical representation of band intensities for the observed mt-transcripts shown in a relative to cells without Dox induction (0 h). Data are the average ± SEM of at least three independent experiments. **P < 0.01; *P < 0.05 (paired t test).

Given that TDP-43 overexpression increased H-strand–encoded mt-tRNA^Leu(UUR)^ (Fig. 4a,b), we also examined whether overexpression of TDP-43 could stabilise H-strand polycistronic transcripts. The H-strand of mtDNA encodes 10 mt-mRNAs for 12 oxidative phosphorylation system subunits (ND1, ND2, COXI, COXII, ATP8-ATP6 (bicistronic), COXIII, ND3, ND4L-ND4 (bicistronic), ND5, and CYB), 2 mt- rRNAs (12S and 16S), and 14 mt-tRNAs (for Phe, Val, Leu (UUR), Ile, Met, Trp, Asp, Lys, Gly, Arg, His, Ser (AGN), Leu (CUN), and Thr) (Supplementary Fig. 2a). To detect transcripts from H-strand mtDNA, we used nine probes for northern blotting (Fig. 5a) and showed that TDP-43 overexpression increased minimally three RNA fragments; those corresponding to 16S-mirrorQ (expected to be 2730 nt, detected by probes 1–4 in Fig. 5a), W-*COXII* (expected to be 2758 nt, detected by probes 6–9 in Fig. 5a) and mirrorANCY-*COXI* (expected to have 1859 nt, detected by probes 7 and 8 in Fig. 5a) (Fig. 5b and c). RT-PCR analysis detected an increase in transcripts containing the boundaries between *ND2* mRNA and mirrorN, and mirrorA and *COXI* mRNA upon overexpression of TDP-43 (Supplementary Fig. 3a), supporting accumulation of mirrorANCY-*COXI* and additional *ND2*-mirrorN fragments in the presence of excess TDP-43. In addition, mt-mRNA quantification using RT-qPCR normalised to *GAPDH* mRNA revealed that TDP-43 overexpression increased transcripts of many of the mt-mRNAs (Supplementary Fig. 3b). Collectively, these data suggested that the increased levels of TDP-43 stabilises at least the region encompassing 16S rRNA to *COXII* mRNA of H-strand transcripts.

Two RNase activities are required for proper mt-tRNA and mt-mRNA maturation: mitochondrial RNase P (MRPP3)-mediated cleavage of precursor molecules at the 5′ end, and tRNase Z (ELAC2)-mediated cleavage at the 3′ end^34^. Therefore, accumulation of large precursor transcripts was also investigated by northern blotting after knockdown of MRPP3 or ELAC2 (Supplementary Fig. 3c and d). The analyses revealed that short interfering RNA (siRNA)-mediated knockdown of MRPP3 but not that of ELAC2 resulted in the accumulation of processing intermediates corresponding to 16S-mirrorQ (detected by probes 1–3), *ND1*-mirrorQ (detected by probes 3 and 4), and W-*COXII* (detected by probes 6–9) relative to 28S rRNA staining compared with a mock knockdown with a sequence-scrambled RNA (scRNA; Supplementary Fig. 3d). All these processing intermediates accumulated in the TDP-43-overexpressing cells (Fig. 5a–c). Thus, the phenotype observed with mitochondrial RNase P deficiency is similar to that observed upon overexpression of TDP-43.

### Overexpression of TDP-43 causes mitochondrial dysfunction

**Figure 6:**
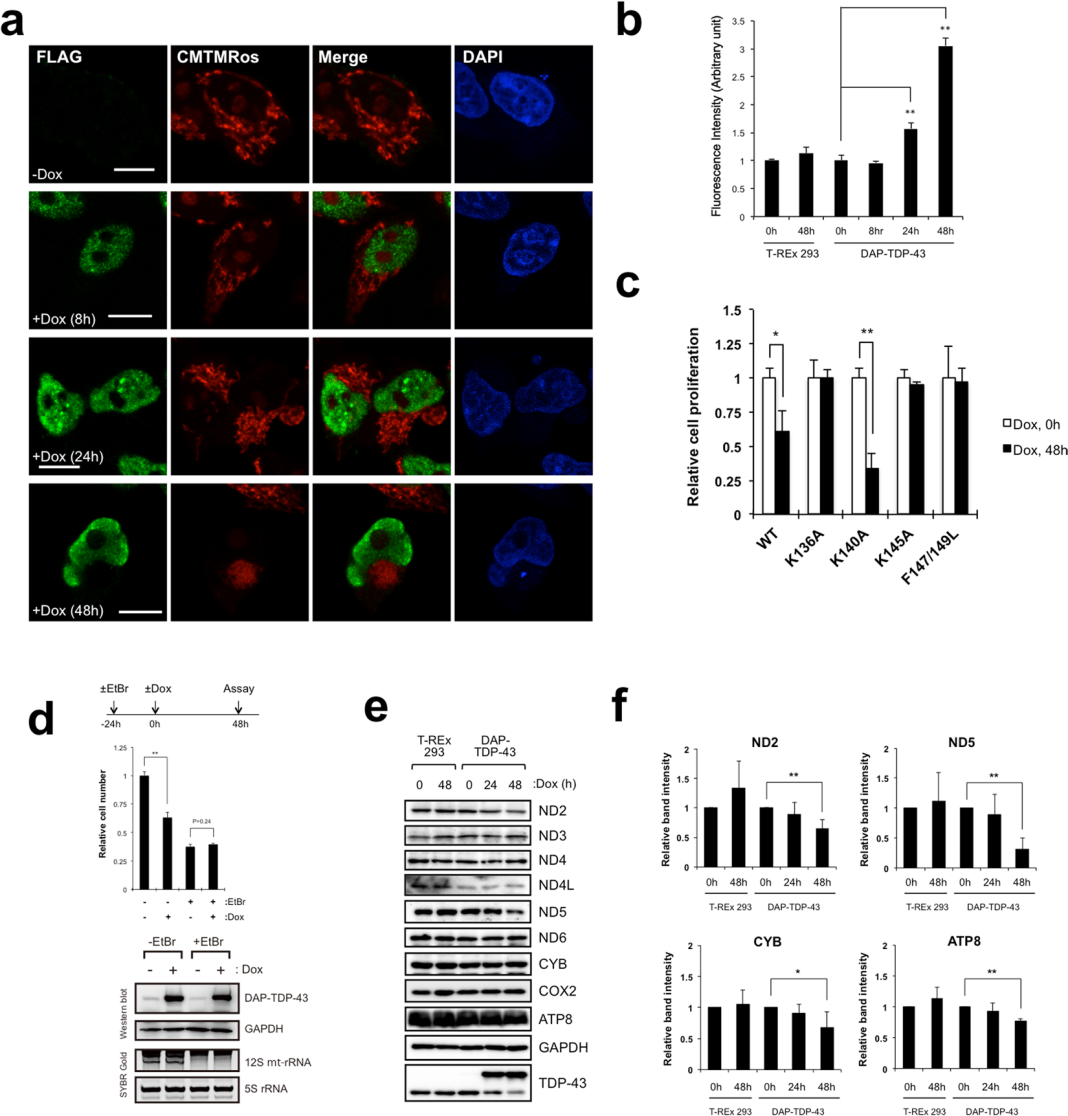
TDP-43 overexpression impairs mitochondrial function. (**a**) Exogenous TDP-43 was detected in DAP-TDP-43–expressing T-REx 293 cells by immunocytochemical analysis with anti-FLAG (green). Mitochondria were stained with CMTMRos (red) and nuclei with DAPI (blue). Cells were stained at 8, 24, or 48 h after doxycycline (Dox) induction. Bars, 10 µm. (**b**) Production of reactive oxygen species was measured in DAP-TDP-43–expressing T-REx 293 cells harvested at 0, 8, 24, and 48 h after Dox induction and in T-REx 293 cells harvested at 0 and 48 h after Dox treatment. The amount of reactive oxygen species (fluorescence intensity; arbitrary unit) per cell is relative to cells without Dox induction. Values are the average ± SD of three independent experiments. **P < 0.01 (unpaired t test). (**c**) Proliferation of T-REx 293 cells expressing DAP-TDP-43 or its point mutants relative to that of cells without Dox induction was measured by cell counting. Values are the average ± SD of three independent experiments. *P < 0.05. **P < 0.01 (unpaired t test). (**d**) DAP-TDP-43-expressing T-REx 293 cells treated with ( + EtBr) or without EtBr (−EtBr) for 24 h were induced with Dox (+Dox) for 48 h, and numbers of cells relative to both −EtBr and −Dox cells were counted. **P < 0.01 (unpaired t test). The expression of DAP-TDP-43 was examined by western blotting with anti-TDP-43 and anti-GAPDH. The inhibition of mt transcription by EtBr was confirmed with SYBR Gold staining of 12S mt-rRNA. (**e**,**f**) Mitochondrial proteins prepared from T-REx 293 cells or from DAP-TDP-43-expressing T-REx 293 cells harvested at 0, 24, and 48 h after Dox induction (Dox) were analysed by western blotting with the indicated antibodies (**e**). Intensities of the immunostained bands in e were quantified relative to those at 0 h of Dox induction. Only the proteins for which the level changed upon overexpression of TDP-43 are shown (**f**). Data are the mean ± SD, n = 3–5. *P < 0.05, **P < 0.01 (paired t test).

Concurrent with the accumulation of processing intermediates, overexpression of TDP-43 in T-REx 293 cells caused irregular staining of mitochondria (Fig. 6a) and led to an increase in amount of reactive oxygen species in cells over time after doxycycline induction (Fig. 6b). Accordingly, TDP-43 overexpression inhibited cell proliferation, whereas the TDP-43 mutants having reduced ability to bind mt-tRNA^Asn^ and mt-tRNA^Gln^ (K136A, K145A, and F147/149L) did not inhibit cell proliferation (Fig. 6c). Notably, we also found that inhibition of mitochondrial transcription with EtBr abolished the inhibitory effect of excess TDP-43 expression on cell proliferation (Fig. 6d). A previous report demonstrated that the defects in the processing of mt-tRNA precursors impair the translation of mtRNAs^35, 36^. To examine whether the cellular level of TDP-43 affects this translation, we estimated the levels of mitochondrial proteins in TDP-43–overexpressing cells and found apparently reduced levels of a number of proteins including ND2, ND5, CYB, and ATP8 at 24–48 h after doxycycline induction (Fig. 6e,f). These results suggested that TDP-43 overexpression deregulates the normal processing of mitochondrial polycistronic transcripts for the production of both tRNAs and mitochondrial proteins. Thus, TDP-43–induced cytotoxicity correlated with mitochondrial functions in human cells as reported for yeast cells expressing human TDP-43^26^.

### TDP-43 is required for maintaining mitochondrial function

**Figure 7:**
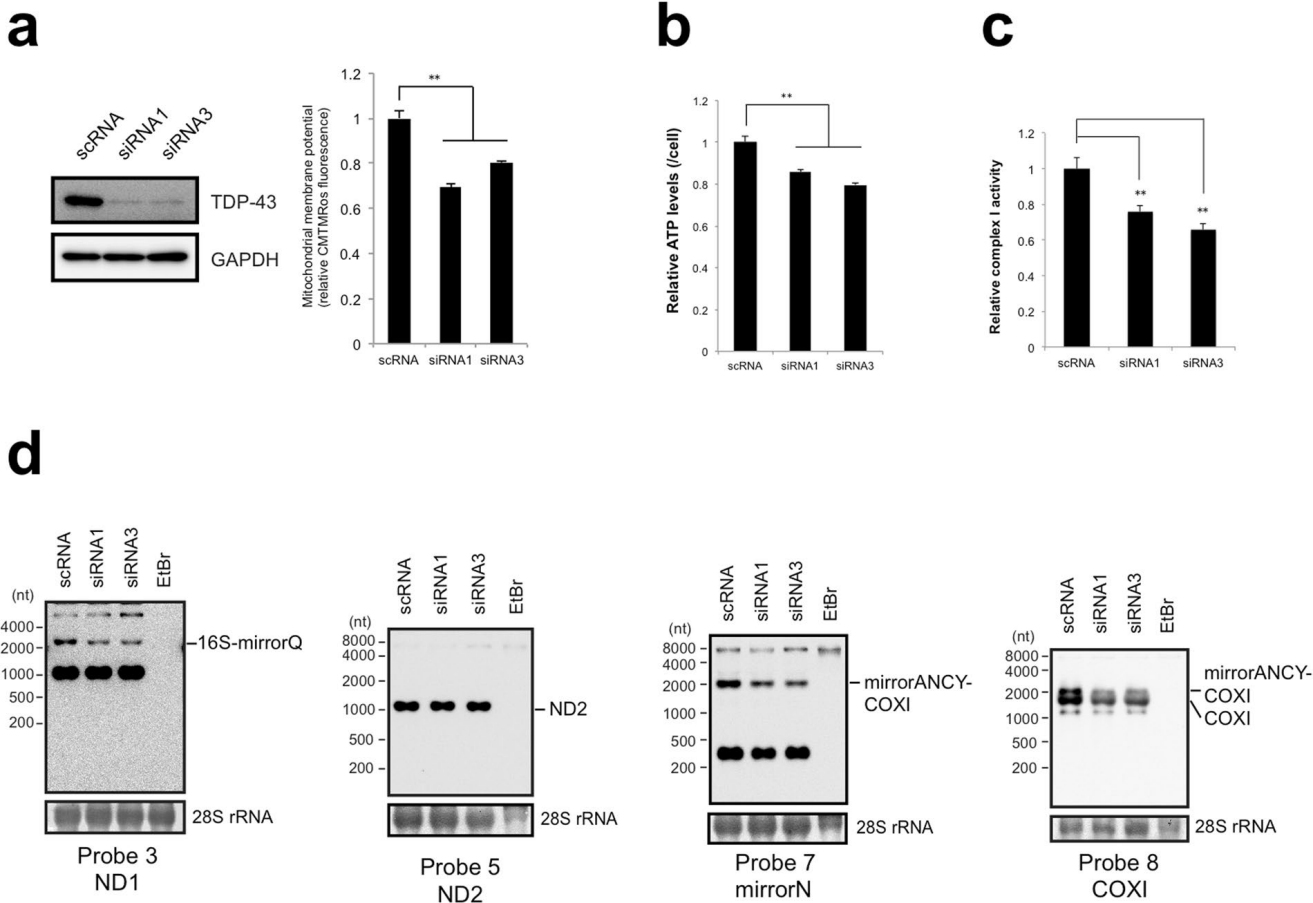
TDP-43 deficiency impairs mitochondrial function and decreases intermediates of mitochondrial transcripts. (**a**) Mitochondrial membrane potential was measured by CMTMRos staining of MCF7 cells after knockdown of TDP-43 with siRNA1 or siRNA3. The membrane potential per concentration of total proteins of the siRNA-treated cells relative to that for scRNA-treated cells harvested at 72 h after treatment is shown. Values are the average ± SD of five independent experiments. **P < 0.01 (unpaired t test). TDP-43 level was examined by western blotting with the indicated antibodies. (**b**) Cellular ATP levels per siRNA-treated MCF7 cell were calculated relative to that for scRNA-treated cells. Values are the average ± SD of six independent experiments. **P < 0.01 (unpaired t test). (**c**) Activity of electron transfer complex I per concentration of total proteins was measured for MCF7 cells harvested at 72 h after siRNA treatment relative to that measured for MCF7 cells with scRNA treatment. Values are the average ± SD of three independent experiments. **P < 0.01. (**d**) mt-tRNA levels upon knockdown of TDP-43 in MCF7 cells were assessed with northern blotting. Probes are indicated under the figures. The intermediates of mitochondrial transcripts were indicated to the right side of the figures. 28S rRNA stained by methylene blue served as a loading controls.

Given the results that TDP-43 affected the processing of mitochondrial polycistronic transcripts and the expression of their processing products, we used several approaches to determine the function of TDP-43 in mitochondria further. We first examined the effects of siRNA-mediated knockdown of TDP-43 on mitochondrial function. TDP-43 knockdown reduced mitochondrial membrane potential and the cellular levels of ATP and the enzyme activity of electron transfer complex I relative to levels in scRNA-treated cells (Fig. 7a–c). In addition, TDP-43 knockdown caused significant reductions on 16S-mirrorQ and mirrorN-*COXI*, as revealed by northern blotting (Fig. 7d). Thus, TDP-43 appears to be required for maintaining appropriate levels of at least certain mt-tRNAs, mt-mRNAs, and mt-mRNA precursors and for maintaining mitochondrial function.

## Discussion

**Figure 8:**
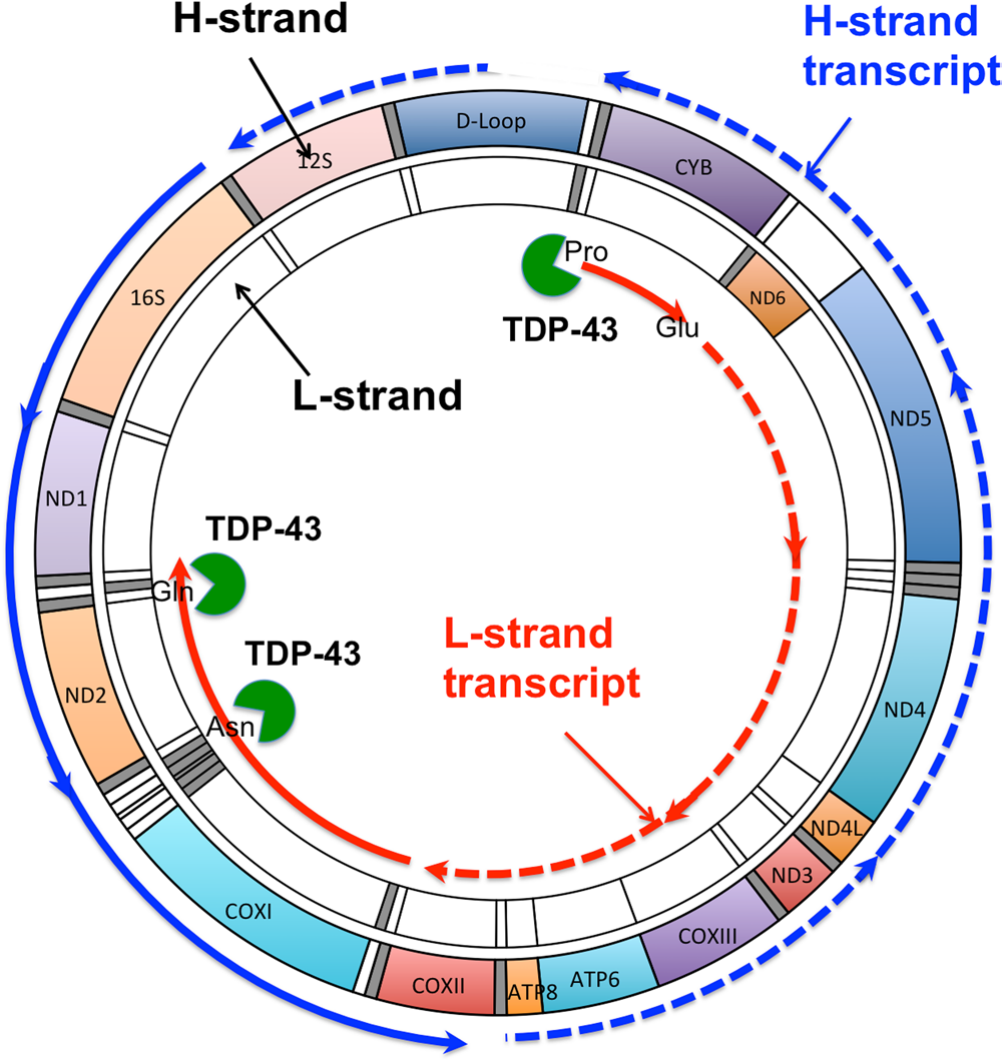
Hypothetical scheme for the effect of increased TDP-43 level on mt polycistronic transcripts. Overexpression of TDP-43 causes an increase in TDP-43 level in mitochondria. The excess TDP-43 stabilises the L-strand transcript (dashed red line) by binding directly to regions corresponding to mt-tRNA^Pro^ (Pro), mt-tRNA^Asn^ (Asn), and mt-tRNA^Gln^ (Gln), and the resulting L-strand-derived transcript (solid red line), and then stabilises one of the transcripts from the H-strand (solid blue line) via complementary double-stand formation. The increased TDP-43 level in mitochondria thereby causes unbalanced processing the mt-transcript, resulting in deregulation of mitochondrial protein synthesis.

In human mtDNA, the genes are located in three different transcription units (one in the L-strand and two in the H-strand) that are transcribed to different extents. The one H-strand transcription unit that includes only the mt**-**rDNA region is transcribed most frequently and is responsible for producing the majority of the mt-rRNAs^37^. The product of the other H-strand transcription unit is a polycistronic RNA molecule corresponding to nearly the entire length of the H-strand that is a precursor for the majority of mt-tRNAs and mt-mRNAs^37^. This H-strand unit is transcribed at ~4% of the frequency of the H-strand mt-rDNA transcription unit^37^. On the other hand, the L-strand unit is transcribed 10–16 times more frequently than the nearly entire H-strand unit^33, 37^. Our present data suggest that TDP-43 overexpression stabilises at least two regions (corresponding to mt-tRNA^Pro^ and mt-tRNA^Asn^/mt-tRNA^Gln^) of this L-strand transcription unit, although we cannot exclude the possibility that the entire L-strand transcript is stabilised by the binding of TDP-43 because the region containing mt-tRNA^Pro^ is located in the first part of the L-strand mtDNA that is transcribed, and the region containing mt-tRNA^Asn^/mt-tRNA^Gln^ is located at the 3′ end. It is also likely that TDP-43 binds to regions of mt-transcripts other than the regions corresponding to the three mt-tRNAs because of its general RNA-binding properties^1, 2^. We also propose that the increase in the L-strand transcript and/or its processing products induced by TDP-43 overexpression may stabilise all or part of the H-strand transcript and/or its processing products through complementary association, resulting in increased levels of these processing products (Fig. 8). Rackham *et al*.^36^ recently reported that mitochondrial MRPP3 is required for the biogenesis of mitochondrial ribosomal subunits that are produced co-transcriptionally on an unprocessed RNA containing the 16S rRNA. Our present data indicate that increased expression of TDP-43 causes accumulation of unprocessed RNA containing the 16S rRNA similar to that caused by conditional knockout of MRPP3^36^. In addition, the results that increased expression of TDP-43 upregulated the levels of mt-transcripts and their products are consistent with those of Rackham *et al*.^36^ in that the conditional knockdown of MRPP3 enhances the rate of transcription and impairs protein synthesis. Moreover, it is also possible that TDP-43 stabilises mt-transcripts and increases the ratio of these to MRPP3, resulting in the accumulation of processing intermediates. Finally, we should note that the unprocessed RNA containing 16S rRNA (indicated as 16S-mirrorQ) seems to be corresponded to RNA 19 that is increased in diseases associated with mt-tRNA^Leu(UUR)^ gene mutations^38, 39^.

Because TDP-43 strictly controls its own expression through a negative feedback loop in which TDP-43 binds to the 3′ untranslated region in its own mRNA^4^, our findings provide important insight into the physiological roles of this negative feedback control and indicate that the strict control of TDP-43 expression is critical for maintaining mitochondrial homeostasis. In this context, mutations in TDP-43 that affect its stability or expression are expected to reduce mitochondrial function. Our present data provides a new mechanism underlying disruption of mitochondrial function by TDP-43 that differs from the mechanisms that involve perturbation of Mfn2^27^, the VAPB–PTPIP51 interaction^29^, or translation of ND3 and ND6^28^.

## Methods

### Immunoprecipitation of DAP-tagged protein associated complex

Confluent cells were washed with PBS and suspended in five packed cell volumes of lysis buffer (50 mM Tris-HCl (pH 7.4), 150 mM NaCl, and 0.5% (w/v) IGEPAL CA-630) containing 2 mM ribonucleoside-vanadyl complex, 1 mM PMSF, 2 µg/ml aprotinin, 2 µg/ml pepstatin A, and 2 µg/ml leupeptin for 30 min on ice. After removal of any insoluble residue by centrifugation at 20,000 × *g* for 30 min at 4 °C, the supernatants were used as cell lysate for the following immunoprecipitation. Cell lysate (5 mg) prepared from 2 × 10^7^ cells were incubated with 15 µl of anti-FLAG M2 magnetic beads (Sigma-Aldrich) for 2 h at 4 °C. The beads were washed five times with 1 ml of lysis buffer, and proteins and RNA were eluted with 150 µl of Protein-RNA extraction buffer (7 M urea, 350 mM NaCl, 1% SDS, 10 mM Tris-HCl (pH 8.0), 10 mM EDTA, and 2% 2-mercaptoethanol) for 5 min. The eluted proteins and RNA were subjected to SDS-PAGE or denaturing (8 M) urea PAGE, respectively, as described previously^40–42^.

### In-gel RNA digestion and LC-MS for RNA analysis

In-gel RNA digestion was performed as described previously^31^. LC-MS analysis of RNA, and database search and interpretation of MS/MS spectra of RNA were performed as described^30, 31, 40, 41^.

### Northern blot analysis

Total RNA was isolated using the RNAgents Total RNA Isolation System (Promega). For analysis of mt-mRNAs, 5 µg of total RNA was subjected to 2% agarose/formaldehyde gel electrophoresis in 3-(N-morpholino)propanesulfonic acid running buffer. For analysis of mt-tRNAs, 1 µg of total RNA was subjected to denaturing (8 M) urea PAGE in Tris-borate-EDTA running buffer, stained with SYBR Gold (Invitrogen) for 10 min, and visualised using the LAS4000 Luminescent Image Analyzer System (Fujifilm). The separated total RNAs were transferred to a Hybond N + membrane (GE Healthcare). The membrane was dried and subsequently cross-linked using an ultraviolet cross linker (Funakoshi, Tokyo) at 120 mJ/cm^2^. For mt-mRNAs analysis, the membranes were stained with methylene blue. Hybridizatioin with biotin-labeled DNA probes and detection of RNA signals were performed as described previously^40, 41^. The oligonucleotides used as probes are shown in Supplementary Table 2.

### UV-CLIP

T-REx 293 cells expressing DAP-TDP-43 were cross-linked by ultraviolet irradiation at 400 mJ/cm^2^, and DAP-TDP-43-associated complexes were immunoprecipitated with anti-FLAG M2 magnetic beads. After heating for 5 min at 65 °C in SDS sample buffer, DAP-TDP-43-associated complexes were released from the anti-FLAG M2 magnetic beads, and separated by SDS-PAGE using a Bis-Tris gel (Running gel: 360 mM Bis-Tis-HCl (pH 6.8), 8% acrylamide, 0.1% tetramethylethylenediamine, 1% ammonium persulfate; Stacking gel: 360 mM Bis-Tis-HCl (pH6.8), 5% acrylamide, 0.1% tetramethylethylenediamine, 1% ammonium persulfate) and Bis-Tris running buffer (50 mM Tris, 50 mM 3-(N-morpholino)propanesulfonic acid, 5 mM EDTA, 0.5% SDS). The separated proteins were electrophoretically transferred to a Protran BA 85 Nitrocellulose membrane (Whatman, Maidstone, UK). The membrane was blocked with 5% non-fat dried skim milk in TBST for 30 min, washed with TBST, and then incubated with HRP-conjugated streptavidin (Thermo Fisher Scientific, San Jose, CA) for 30 min. After washing three times with TBST for 10 min, the Chemiluminescent Nucleic Acid Detection Module kit (Thermo Fisher Scientific) was used for detecting biotin of DAP-TDP-43. Stained parts of the membrane were excised and incubated in 250 µl of Proteinase K buffer (10 mM Tris-HCl pH 8.0, 30 mM NaCl, 10 mM EDTA, 2 mg/ml Proteinase K) at 37 °C for 30 min. After addition of 250 µl of Protein-RNA extraction buffer, RNA was isolated by phenol-chloroform extraction and isopropanol precipitation and subjected to northern blot analysis.

### RNA Synthesis by *in vitro* transcription

The template DNAs for *in vitro* transcription of mt-tRNA^(Asn)^, AC-stem(Leu), D-Loop(Leu), pAntiCdn(Leu), Var-R(Leu), T-Loop(Leu), and Leu(UUR) RNA were generated by annealing the primer sets shown in Supplementary table 2, respectively. *In vitro* transcription was performed with 0.2 pmol of the template DNA using CUGA® 7 *in vitro* transcription kit (NIPPON GENE) according to the manufacturer’s instructions. Transcripts were collected by isopropanol precipitation, and purified using reverse-phase liquid chromatography. To synthesize biotinylated mirrorCOXI-YCNA RNA, template DNA for *in vitro* transcription was prepared. We amplified DNA fragments corresponding to mtDNA between mirror mt-tRNA^Asn^ to COX I with the primer set (mirrorANCY-COXI-for/ mirrorANCY-COXI-for) using KOD neo plus (TOYOBO), and inserted them into the BamHI/PstI site of the pSPT19 vector (Roche) (mirrorANCY-COXI pSPT19). mirrorANCY-COXI pSPT19 linearlized by digesting with SmaI was used as template DNA for *in vitro* transcription. Biotin-labeled mirrorCOXI-YCNA was *in vitro* transcribed from the DNA template (0.06 pmol) in the presence of 7.5 mM of ATP, 7.5 mM of CTP, 7.5 mM of UTP, 7.5 mM GTP, 0.5 mM of biotin-UTP (Roche) using CUGA® 7 *in vitro* transcription kit (NIPPON GENE, Japan), and purified by 1% agarose gel electrophoresis and the subsequent gel extraction using Zymoclean Gel RNA Recovery Kit (ZYMO RESEARCH).

### EMSA

Each binding reaction was performed at room temperature for 20 min. Each reaction was performed in 10 µl binding buffer (40 mM Tris-HCl (pH 7.4), 30 mM KCl, 1 mM MgCl_2_, 0.01% IGEPAL CA-630, 1 mM DTT, 10 µg of yeast tRNA (Ambion), 10 µg BSA, 10 ng of synthetic RNA, 200 ng of recombinant protein), and electrophoresed on a 6% non-denaturing polyacrylamide gel at 100 V for 50 min in 0.5× Tris borate/EDTA buffer (44.5 mM Tris-borate and 1 mM EDTA). The separated RNA-protein complexes were transferred to a Hybond N+ membrane that was dried and UV-crosslinked using the FS-1500 crosslinker (Funakoshi) at 120 mJ/cm^2^. The synthetic RNA was detected by northern blot analysis using the indicated probes.

### Competition Binding Assay with synthetic RNA

Total cell lysate from 1.0 × 10^7^ cells expressing DAP-TDP-43 was used for this assay. Synthetic RNA (sRNA) (1 pmol) was added to the total cell lysate and subjected to immunoprecipitation using anti-FLAG magnetic beads as described above. RNA isolated from DAP-TDP-43 complex was subjected to denaturing urea-PAGE and northern blot analysis.

### Mitochondria Isolation

Mitochondria were prepared from T-REx 293 cells or 293 T cells grown in 5 × 15 cm^2^ dishes. Cells washed twice with PBS were sedimented (800 × g for 5 min at 4 °C), and resuspended in 9 ml of ice-cold Solution A (20 mM Hepes-KOH pH7.6, 220 mM Mannitol, 70 mM Sucrose, 1 mM EDTA, 1 mM PMSF, 2 mg/ml fatty acid free BSA). Cells were homogenized by passing ten times with a 25 G needle. The nuclei from this suspension were sedimented (800 × g for 10 min at 4 °C), and the centrifugation step was repeated for the supernatant once more. Mitochondria were sedimented from the supernatant by centrifugation (15,000 × g for 20 min at 4 °C), and washed once with 1 ml of Solution A. Protein concentration was measured using NanoDrop. For Proteinase K protection assay, the isolated mitochonaria was diluted into 1 mg/ml with Solution B (10 mM Hepes-KOH pH7.6, 500 mM Sucrose). 100 µg of mitochondria was treated with the indicated concentration of Proteinase K (Takara Bio, Osaka, Japan) for 20 min on ice. Mitochondria were treated with 0.5% (w/v) of Triton X-100 along with the treatment of Proteinase K for a control of Proteinase K protection assay. Proteinase K reaction was stopped with the addition of 1 mM PMSF, and the mitochondria were centrifuged (12,000 × g for 5 min at 4 °C), resuspended with SDS sample buffer, and subjected to western blot analysis.

### Preparation of Mitoplast extraction

100 µg of Mitochondria isolated from 293 T cells were resuspended with 500 µl of 20 mM Hepes-KOH pH 7.4, and incubated for 20 min on ice. After sedimenting mitochondria by centrifugation (10,000 × g for 10 min at 4 °C), pellet were washed once with Solution B, resuspended with 50 µl of 20 mM Hepes-KOH pH 7.4, and disrupted twice for 30 sec using Bioruptor UCD-250 (Cosmo Bio). After sedimenting them by centrifugation (20,000 × g for 30 min at 4 °C), the supernatants were used as a mitoplast extraction. Protein concentration was measured using BCA assay.

### mtRNA degradation assay

mtRNA degradation assay was performed at 25 °C for 5, 10 or 30 min. Each reaction was performed in 20 µl of reaction buffer (20 mM Tris-HCl (pH 7.4), 60 mM KCl, 12.5 mM MgCl2, 0.1 EDTA, 2 mM DTT, 10% Glycerol, 10 µg mitoplast extract, 10 fmol biotinylated mirrorCOXI-YCNA RNA, 600 ng TF-fused protein). Reaction was stopped by the addition of 150 µl Protein-RNA extraction buffer (7 M urea, 350 mM NaCl, 1% SDS, 10 mM Tris-HCl (pH 8.0), 10 mM EDTA, and 2% 2-mercaptoethanol), and protein or RNA in the reaction was purified as described previously^40, 41^. The eluted proteins and RNA were subjected to SDS-PAGE or northern blot analysis using 1.5% denaturing agarose gel. Biotinylated RNA was detected using HRP-conjugated Streptavidine.

## Electronic supplementary material

Supplementary Information
