# Supplementary material for: TDP-43 stabilises the processing intermediates of mitochondrial transcripts
Source: Sci Rep. 2017 Aug 9;7:7709. doi: 10.1038/s41598-017-06953-y (PMC5550480; doi:10.1038/s41598-017-06953-y)

Supplementary information

TDP-43 stabilises the processing intermediates of mitochondrial transcripts

Keiichi Izumikawa[1, 2], Yuko Nobe[2, 3], Harunori Yoshikawa[1, 2, 4], Hideaki Ishikawa[1, 2], Yutaka Miura[1], Hiroshi Nakayama[5], Takashi Nonaka[6], Masato Hasegawa[6], Naohiro Egawa[7], Haruhisa Inoue[7], Kouki Nishikawa[8], Koji Yamano[9], Richard J. Simpson[1, 10], Masato Taoka[2, 3], Yoshio Yamauchi[2, 3], Toshiaki Isobe[2, 3], and Nobuhiro Takahashi[1, 2]*

[1] Global Innovation Research Organizations, Tokyo University of Agriculture and Technology, 3-5-8 Saiwai-cho, Fuchu, Tokyo 183-8509, Japan

[2] Core Research for Evolutional Science and Technology (CREST), Japan Science and Technology Agency (JST), Sanbancho 5, Chiyoda-ku, Tokyo 102-0075, Japan

[3] Department of Chemistry, Graduate School of Science, Tokyo Metropolitan University, 1-1 Minami-ohsawa, Hachioji, Tokyo 192-0397, Japan

[4] Centre for Gene Regulation & Expression, School of Life Sciences, University of Dundee, Dow Street, Dundee, DD1 5EH, UK

[5] Biomolecular Characterization Unit, RIKEN Center for Sustainable Resource Science, 2-1 Hirosawa, Wako, Saitama 351-0198 Japan

[6] Department of Dementia and Higher Brain Function/Dementia Research Project Tokyo Metropolitan Institute of Medical Science 2-1-6 Kamikitazawa Setagaya-ku Tokyo 156-8506 Japan

[7] Center for iPS Cell Research and Application (CiRA), Kyoto University, Kyoto 606-8507, Japan

[8] Cellular and Structural Physiology Institute (CeSPI) Nagoya University Nagoya 464-8601 Japan

[9] Ubiquitin project Tokyo Metropolitan Institute of Medical Sciences Setagaya-ku Tokyo 156-8506 Japan

[10] La Trobe Institute for Molecular Science (LIMS), LIMS Building 1, Room 412 La Trobe University, Bundoora Victoria 3086, Australia

Materials and Methods

Antibodies and Reagents

Antibodies used in this study were: Mouse monoclonal anti-TDP-43 (ProteinTech, Chicago, IL; 60019-2-Ig), rabbit polyclonal anti-TDP-43 (ProteinTech; 10782-2-AP), mouse monoclonal anti-FLAG (Sigma-Aldrich, St Louis, MO; M2), nonspecific rabbit IgG (Abcam, Cambridge, MA; ab46540), FITC-conjugated anti-mouse IgG (KPL, Gaithersburg, MD), mouse monoclonal anti-GAPDH (Ambion, Austin, TX; AM4300), rabbit polyclonal anti-ND2 (Abgent, San Diego, CA; AP9387a), rabbit polyclonal anti-ND3 (Abgent; AP12310a), rabbit polyclonal anti-ND4 (Santa Cruz Biotechnology, Santa Cruz, CA; sc-20499R), rabbit polyclonal anti-ND4L (Santa Cruz Biotechnology; sc-20665), rabbit polyclonal anti-ND5 (Abgent; AP6939b), rabbit polyclonal anti-ND6 (Lifespan Biosciences, Seattle, WA; LS-C82589-50), rabbit polyclonal anti-CYB (Abgent; AP9621C), mouse monoclonal anti-COX II (Abcam; ab110258), rabbit polyclonal anti-ATP8 (Santa Cruz Biotechnology; sc-84231), rabbit polyclonal anti-ELAC2 (Proteintech; 10071-1-AP), rabbit anti-MRPP3 (GeneTex; GTX121658), anti-TFAM (BETHYL; A303-226A), anti-TOM22 (GTX10436), horseradish peroxidase (HRP)-conjugated anti-mouse IgG (Cell Signaling Technology, Beverly, MA, #7076), and HRP-conjugated anti-rabbit IgG (Cell Signaling Technology, Beverly, MA, #7074). For the detection of biotinylated oligonucleotide, we used Stabilized Streptavidin-HRP Conjugate (Thermo Scientific, 89880D). All general reagents were purchased from Wako Pure Chemical, Osaka, Japan; Kanto Chemical Co., or Nacalai Tesque.

Cell culture

HeLa, MCF7, 293T (HEK293 cells transformed with large T antigen) and Flp-In T-REx 293 cells were cultured as described previously[1,2]. Cells were treated with EtBr (Invitrogen, Carlsbad, CA) at 250 ng/ml for the indicated time periods.

Construction of epitope-tagged protein expression vectors

To construct the FLAG-tagged TDP-43 expression vector, the TDP-43 DNA fragment was amplified by from pEGFP-TDP-43 prepared by Arai *et al*[3] with KOD-plus DNA polymerase (Toyobo, Osaka, Japan). The PCR product was

cloned into the *KpnI/XhoI* sites of pcDNA5/FRT/TO (Invitrogen); the resulting vector was designated as FLAG-TDP-43 pcDNA5-FRT/TO. A triple affinity-tag (DAP) used for the inducible TDP-43 expression cell line was constructed by PCR amplification of a cDNA encoding 6 histidines and a biotinylated sequence using the vector pcDNA3.1(+)-bio as a template based on the method described previously[4]. The PCR product was cloned into the HindIII/KpnI sites of FLAG-TDP-43 pcDNA5-FRT/TO; the resulting vector was designated as DAP-TDP-43 pcDNA5/FRT/TO. To construct Δ RRM1, Δ RRM2, or Δ GR, DNA fragments were amplified using KOD plus DNA polymerase with the primer sets (Δ RRM1; ClaI-TDP43(170-)-For/TDP43(-104)-ClaI-Rev, Δ RRM2; ClaI-TDP43(258-)-For/TDP43(-192)-ClaI-Rev, Δ GR; ClaI-TDP43(315-)-For/TDP43(-273)-ClaI-Rev), digested with ClaI, and subjected to the self-ligation. To construct Δ 315, DNA fragments were amplified by KOD plus DNA polymerase with the primer set (BamHI-TDP43-1-For/TDP43(-314)-XhoI-Rev), digested with BamHI/XhoI, and inserted into the BamHI/XhoI site of DAP-tag pcDNA5/FRT/TO that was generated by exciting TDP-43 fragment with BamHI/XhoI from DAP-TDP-43 pcDNA5/FRT/TO. To construct K136A, K140A, K145A, or F147/149L, DNA fragments were amplified using KOD plus DNA polymerase with the primer sets (K136A; K136A-For/K136A-Rev, K140A; K140A-For/K140A-Rev, K145A; K145A-For/K145A-Rev, F147/149L; F147/149L-For/ F147/149L-Rev), and template DNA was degraded with DpnI. Construction of DAP-Fibrillarin pcDNA5FRT/TO was described before[5]. To construct pCold-TF-FLAG, the primer set (pCold-FLAG-For/ pCold-FLAG-Rev) was annealed, digested with HindIII/XbaI, and inserted into the HindIII/XbaI site of pCold-TF vector (Clontech). To construct pCold-TF-TDP43-FL, DNA fragments were amplified using KOD plus DNA polymerase with the primer set (BamHI-TDP43-HindIII-For/ BamHI-TDP43-HindIII-Rev), digested with BamHI/HindIII, and inserted into the BamHI/HindIII site of pCold-TF-FLAG. All constructs were verified by DNA sequencing. The primer sets used to amplify each gene are shown in Supplementary Table 2.

Construction of doxycycline-inducible cell lines

The cell lines expressing doxycycline inducible DAP-tagged protein was established as described previously[1,2]. To induce expression of the DAP- tagged protein, 100 ng/ml of doxycycline was treated to the cells for the indicated time periods.

Immunoprecipitation of endogenous TDP-43 complex

Rabbit polyclonal anti-TDP-43 antibody (3 µg) (ProteinTech; 10782-2-AP) was bound to Dynabeads by incubating with 15 µl of protein G Dynabeads (Invitrogen) in PBS containing 0.01% Triton X-100 for 30 min at 25°C and then washing three times with PBS containing 0.01% Triton X-100. The antibody-bound Dynabeads were mixed with 2 mg of total cell lysate (prepared from 1.0 x 10^7 cells) for 4 h at 4°C and washed five times with 1 ml of lysis buffer. Proteins and RNA were recovered from the antibody-bound Dynabeads as described above. (See Method section for Immunoprecipitation of DAP-tagged protein associated complex).

Immunoblot analysis

Immunoblot analysis was performed as described[1,2]. Signal intensity of each band was measured and quantified using MultiGauge software (Fujifilm, Tokyo, Japan).

Immunoelectron microscopy

Cells were fixed with 4% paraformaldehyde and 0.1% glutaraldehyde in PBS and harvested by centrifugation for 5 min at 4°C at 200 x g, followed by emersion in 55°C 2% agar. After cooling, the cell-containing agar was cut into 1 mm square blocks. The blocks were immersed in 2% uranyl acetate dissolved in water for contrast and dehydrated by immersion in graded concentrations of ethanol (50, 60, 70, 80, 90, and 100%). The temperature was lowered by 10°C in each methanol step from 0°C to −50°C in a cryo-substitution unit (Leica EM AFS). The samples were infiltrated with Lowicryl HM20 resin (Polysciences, Inc., Warrington) at –50°C with a progressive increase in the ratio of resin to ethanol (1:2, 1:1, 2:1). Finally, the samples were incubated in pure resin overnight at −

50°C. Polymerization was carried out under ultraviolet light for 48 h at −50°C and for 24 h at room temperature. Samples were conventionally sectioned (70 nm thickness) with the Leica EM UC6 microtome. The ultrathin sections were treated with the following solutions: (1) 5% BSA, 0.005% saponin and 0.001% sodium azide in PBS to prevent non-specific reactions; (2) primary antibodies against TDP-43 (ProteinTech 60019-2-Ig; 50 μg/ml or 10 μg/ml) in PBS containing 1% BSA, 0.005% saponin and 0.001% sodium azide for 3 h at room temperature and overnight at 4°C; and (3) 15 nm gold-conjugated secondary antibody (BBInternational; 1:100) in PBS containing 1% BSA, 0.005% saponin and 0.001% sodium azide for 3 h at room temperature. The sections were negatively stained with 2% uranyl acetate and 2% lead acetate. Electron micrographs were recorded on a JEM-1010 (Jeol) transmission electron microscope equipped with a 2K CMOS camera (TVIPS TemCam F216) at 2,500 and 8,000× magnification and operated at 100 kV. TDP-43 was visualised by 15 nm immunogold particles.

RT-PCR analysis

Total RNA was treated with Baseline-ZERO DNase (Epicentre Biotechnologies, Madison, WI) for 1 h at 37°C, and purified using the RNAgents Total RNA Isolation System (Promega). For RT-PCR analysis, the DNase treated total RNA was reverse-transcribed with gene specific primers using the PrimeScript RT-PCR kit (Takara Bio, Japan). PCR was performed using TaKaRa Ex Taq Hot Start Version (Takara Bio), and the products were separated in an 8% polyacrylamide gel (1× Tris-borate-EDTA) or 1.5% agarose gel (0.5× Tris-acetate-EDTA). The polyacrylamide gels were stained with SYBR Gold and the agarose gels with EtBr. The amplified genes were visualised using the LAS4000 Luminescent Image Analyzer System and MultiGauge software (Fujifilm). For RT-qPCR analysis, the DNase treated total RNA was reverse transcribed with random hexamers and oligo dT primers using the PrimeScript RT Reagent kit (Takara Bio, Japan). For ND6, specific primers were used in the reverse transcription. qPCR was performed using SYBR Premix Ex Taq II (Takara Bio) and the Thermal Cycler Dice Real Time System (Takara Bio).

Relative gene expression was determined by the ΔΔCT method and the quantified values were normalised to the values obtained for GAPDH mRNA. The primer sets used to amplify each gene are shown in Supplementary Table 2.

Purification of TF-fused Protein

TF-TDP-43-FL (TF-TDP-43) or TF-FL was expressed in Escherichia coli Rosetta2 (DE3) via a 24 h induction at 15°C by the presence of 0.1 mM Isopropyl β -D-1-thiogalactopyranoside. Escherichia coli expressing TF-TDP-43-FL or TF-FL was lysed with xTractor Buffer (Clontech), and TF-fused proteins were purified using Ni-NTA agarose (Qiagen), and eluted with 250 mM imidazole in 50 mM Tris-HCl pH7.4 and 150 mM NaCl. The eluted TF-fused proteins were isolated by ANTI-FLAG M2 Affinity Agarose Gel (SIGMA), and eluted with FLAG peptide. The eluted solution containing TF-fused proteins were dialyzed with Tris-buffered saline for 12 h at 4°C, and quantified with the BCA protein assay (Thermo Scientific).

RNA interference.

Stealth siRNA1, siRNA3 or scRNA (negative control) (Invitrogen) transfection was performed using Lipofectamine RNAiMax (Invitrogen). In 35-mm Petri dishes, HeLa and MCF7 cells were transfected with 50 pmol of scRNA or siRNA, and 293T cells were transfected with 100 pmol of scRNA or siRNA. The siRNA1, siRNA3 and scRNA sequences are shown in Supplementary Table 2.

Immunocytostaining.

Immunocytostaining was performed as described previously[1,2,4]. Cells were cultured in the wells of collagen-coated culture slides (BD-Falcon Biosciences, Lexington, TN) and incubated for 20 min with 100 nM MitoTracker Orange CMTMRos (Invitrogen) for mitochondria staining before the cells were fixed. Fluorescence of the cells was visualised with an Axiovert 200 M microscope (Carl Zeiss, Oberkochen, Germany).

Measurement of ROS production.

Intracellular ROS levels were assessed by flow cytometric analyses using a

fluorometric probe (2’,7’-dichlorofluorescein diacetate; Molecular Probes, Eugene, OR) with the EPICS Elite EPS cell sorting system (Beckman-Coulter, Hialeah, FL) as described previously[6].

Cell proliferation assay

To assese cell proliferation cell number was calculated visually using a Burker-Turk chamber (Hirschmann, Laborgeräte Hilgenberg, Germany).

Measurement of mitochondrial membrane potential

Cells were cultured for 20 min with 100 nM MitoTracker Orange CMTMRos (Invitrogen) and then washed with ice-cold PBS. Luminescence was measured using the GloMax-Multi Detection System (Promega). The luminescence detected was normalised to the protein content of each sample measured by Pierce BCA assay (Thermo Fisher Scientific).

Measurement of cellular ATP levels

Cells were harvested and washed with ice cold PBS. ATP levels in the cells were measured using the CellTiter-Glo Luminescent Cell Viability Assay (Promega). The luminescence was detected with the LAS4000 Luminescent Image Analyzer System and quantified by MultiGauge software (Fujifilm). Signal intensity was normalised to the cell number of each sample.

Measurement of complex I enzyme activity

Mitochondrial complex I enzyme activity was measured using the Complex I Enzyme Activity Dipstick Assay Kit (Abcam). The Signal intensities were measured with the LAS4000 system and quantified by MultiGauge software (Fujifilm).

Supplementary Figure 1. TDP-43 associates with a subset of L-strand-encoded mt-tRNAs.

a, Schematic diagram of a tandem affinity tag (DAP-tag). **b**, T-REx 293 cells inducibly expressing DAP-TDP-43 treated with (+) or without (-) doxycycline for 48 h, and endogenous TDP-43 was detected by immunobot using anti-TDP-43. Immunoblot using GAPDH served as loading controls. **c**, RNA was immunoprecipitated (IP) with anti-FLAG from T-REx 293 cells inducibly expressing DAP-TDP-43 or DAP-fibrillarin, and analysed by northern blotting (NB) with probes indicated on the right. DAP-TDP-43 and DAP-fibrillarin were detected by western blotting (WB) using HRP-conjugated streptavidin. T-REx 293 cells served as a negative control, and DAP-fibrillarin served as a control for DAP-tag. **d**, Schematic representation of DAP-TDP-43 and its deletion or point mutants. RRM, RNA recognition motif; G-rich, glycine rich. **e**, RNA immunoprecipitated using anti-FLAG from T-REX 293 cells expressing DAP-TDP-43 deletion mutants was analysed by NB with the indicated probes on the right. DAP-tagged proteins were detected by WB using HRP-conjugated streptavidin. **f**, Nucleotide sequences of the synthesised mt-tRNA^{Asn} and its mutants; the sequences in red were replaced by sequences corresponding to the region of acceptor stem [Ac-stem(Leu)], D-loop(Leu), anticodon loop [pAntiCdn(Leu)], variable region [Var-R(Leu)], or T-loop(Leu) in mt-tRNA^{Leu}. The sequence of the synthesised mt-tRNA^{Leu} [Leu(UUR)] is also shown. The residue numbers of the nucleotide sequence are shown above. Lines with arrows indicate DNA probes (Asn1 and Asn2) corresponding to the sequence used. **g**, Purified recombinant trigger factor (TF, 500 ng), TF-fused TDP-43 (TF-TDP-43, 500 ng), and bovine serum albumin (BSA, 250, 500, 1000 ng) were separated by SDS-PAGE and visualised by Coomassie Brilliant Blue staining (left). The synthesised mt-tRNA^{Asn} or mt-tRNA$^{Leu(UUR)}$ was mixed with TF-TDP-43, analysed by electrophoretic mobility shift assay, and detected by NB with probe Asn1 and Leu that is complementary to mt-tRNA^{Leu}(UUR). NT indicates the absence of recombinant protein.

Supplementary Figure 2. Mitochondria transcription was inhibited by ethidium bromide.

a, Schematic diagram of mt-rRNA, mt-tRNA, and mt-mRNA encoded in the mitochondrial genome. The H-strand and L-strand of mtDNA are indicated. **b, c**, T-REx 293 cells were treated with EtBr to inhibit mitochondrial transcription for the indicated periods. Total RNA separated on a denaturing urea gel (b) or agarose gel (c) were analysed by northern blotting with the probes indicated on the right. 28S rRNA stained with methylene blue were used as loading controls.

Supplementary Figure 3. MRPP3 deficiency causes accumulation of intermediates of mitochondrial transcript processing similar to that observed after TDP-43 overexpression.

a, RNA regions corresponding to the boundary between ND2 mRNA and mirrorN and the mirrorA and COXI mRNAs were detected by RT-PCR. PCR fragments were separated on an agarose gel and stained with EtBr. Size standards (bp) are shown to the left. 5S rRNA and ACTB mRNA served as loading controls. **b**, Total RNA extracted from DAP-TDP-43–expressing T-REx 293 cells harvested at 0, 24, or 48 h after Dox induction were reverse transcribed and analysed by real-time quantitative PCR. The mt-mRNA levels measured by quantitative RT-PCR are graphed. Both exogenous and endogenous TDP-43 mRNA (TDP-43 code) and endogenous TDP-43 mRNA (TDP-43 3' untranslated region, UTR) were measured as controls for exogenous TDP-43 expression. Values were normalised to GAPDH mRNA. Data are the average ± SD, n = 3–8. **P < 0.01; *P < 0.05 (paired t test between values with (24 or 48 h) and without (0 h) Dox induction). **c**, 293T cells in which ELAC2 or MRPP3 was knocked down with siRNA were analysed by western blotting with the indicated antibodies on the right. **d**, Total RNA extracted from ELAC2 or MRPP3 knocked-down 293T cells (treated with siRNA/scRNA for 48 h) was analysed by northern blotting with the probes indicated under the figures. EtBr served as a negative control for the mitochondrial transcripts. The processing intermediates of mitochondrial transcripts identified from the results of northern blotting are indicated on the right side of figures. +16S-mirrorQ; *W-COXII; **mirrorANCY-COXI. 28S and 18S rRNA stained with Methylene blue served as loading controls.

1 Izumikawa, K. *et al.* Friend of Prmt1, FOP is a Novel Component of the Nuclear SMN Complex Isolated Using Biotin Affinity Purification. *Journal of Proteomics & Bioinformatics* **07**, doi:10.4172/jpb.S7-002 (2014).

2 Ishikawa, H. *et al.* Identification of truncated forms of U1 snRNA reveals a novel RNA degradation pathway during snRNP biogenesis. *Nucleic Acids Res* **42**, 2708-2724, doi:10.1093/nar/gkt1271 (2014).

3 Arai, T. *et al.* TDP-43 is a component of ubiquitin-positive tau-negative inclusions in frontotemporal lobar degeneration and amyotrophic lateral sclerosis. *Biochem Biophys Res Commun* **351**, 602-611, doi:10.1016/j.bbrc.2006.10.093 (2006).

4 Miyazawa, N. *et al.* Human cell growth regulator Ly-1 antibody reactive homologue accelerates processing of preribosomal RNA. *Genes Cells* **19**, 273-286, doi:10.1111/gtc.12129 (2014).

5 Sato, S. *et al.* Collaborator of alternative reading frame protein (CARF) regulates early processing of pre-ribosomal RNA by retaining XRN2 (5'-3' exoribonuclease) in the nucleoplasm. *Nucleic Acids Res* **43**, 10397-10410, doi:10.1093/nar/gkv1069 (2015).

6 Yagihashi, S., Miura, Y. & Yagasaki, K. Inhibitory effect of gingerol on the proliferation and invasion of hepatoma cells in culture. *Cytotechnology* **57**, 129-136, doi:10.1007/s10616-008-9121-8 (2008).

Supplementary Table 1. Identification of RNase T1 fragments of mt tRNAs in the TDP-43 ribonucleoprotein complex.

Observed			Theoretical							MS/MS fragment ions detected				
m/z	charge	Molecular mass (Da)	Molecular mass (Da)	error (ppm)	Rt	RNA	Residue numbers	Sequence	Idenitificatio n method[a]	a series	c series	w series	y series	Remarks
691.1027	1	692.1105	692.1105	0.0	29.71	mt $tRNA^{Pro}$	4-5, 48-49	AGp	M	2	1		1	
834.1209	2	1670.2575	1670.2565	0.6	36.57	mt $tRNA^{Pro}$	6-10	AAU(mA)Gp	A	3,4,5	1,2,3,4	1,3,4,	1,2,3,4	Bsae loss of monomethylated adenine was detected in the MS/MS spectrum.
1068.7909	3	3209.3961	3209.3946	0.5	38.62	mt $tRNA^{Pro}$	11-20	UUUAAAUUAGp	A	4,8,10	2,3,4,5,6, 7,8,9	3,4,5,6	1,2,3,4,5,6, 7,8,9	
1285.6598	2	2573.3352	2573.3328	1.0	38.45	mt $tRNA^{Pro}$	21-28	AAUCUUAGp	A	4,8	2,3,4,5,6, 7	3,4,6,7	2,3,4,5,6,7	
792.0803	2	1586.1762	1586.1752	0.6	28.21	mt $tRNA^{Pro}$	29-33	CUUUGp	A	2,3,5	1,2,3,4	1,2,3,4	1,2,3,4	
792.0804	2	1586.1764	1586.1752	0.8	29.30	mt $tRNA^{Pro}$	29-33	CUUUGp	A	3,5	2,3,4	1,2,4	1,2,3,4	
1027.1400	1	1028.1478	1028.1464	1.4	30.11	mt $tRNA^{Pro}$	35-37	(mG)UGp	M	2,3	1,2	1,2	1,2	Bsae loss of monomethylated guanine was detected in the MS/MS spectrum.
968.1210	2	1938.2577	1938.2549	1.4	32.70	mt $tRNA^{Pro}$	38-43	CUAAUGp	A	4,6	2,3,4,5	3,5	1,2,3,4,5	
987.1333	2	1976.2823	1976.2818	0.2	35.33	mt $tRNA^{Pro}$	50-55	(mU)UAAAGp	A	2,6	2,3,4,5	1,2,3,4	1,2,3,4,5	
980.1260	2	1962.2676	1962.2662	0.7	33.87	mt $tRNA^{Pro}$	50-55	UUAAAGp	M	2,6	2,3,4,5		1,2,3,4,5	
980.1259	2	1962.2674	1962.2662	0.6	35.10	mt $tRNA^{Pro}$	50-55	UUAAAGp	A	6	2,3,4,5	2	1,2,3,4,5	
1248.7969	3	3749.4141	3749.4115	0.7	34.95	mt $tRNA^{Pro}$	56-67	ACUUUUUCUCUGp	A	4,12	2,3,6,8,9, 11	2,4	2,3,4,5,6,7, 9,10,11	
1248.7964	3	3749.4126	3749.4115	0.3	35.85	mt $tRNA^{Pro}$	56-67	ACUUUUUCUCUGp	A	5,12	2,3,5,6,7, 8,9,10	2,4,9,11	2,3,4,5,6,7, 9,10,11	
602.1092	2	1206.2340	1206.2318	1.8	36.81	mt $tRNA^{Pro}$	68-71	ACCA-OH	M	2,3	2,3	2	2,3	
1077.0924	1	1078.1002	1078.1022	-1.8	26.06	mt $tRNA^{Asn}$	1-3	pUAGp	M	2	1,2	1,2	2,3	
651.0730	2	1304.1617	1304.1611	0.4	35.37	mt $tRNA^{Asn}$	4-7	AUUGp	A	2,3,4	1,2,3	2,3	1,2,3	
1048.1854	1	1049.1932	1049.1943	-1.0	37.01	mt $tRNA^{Asn}$	8-10	A(mA)(mG)p	M	3	2	1	1,2	Bsae losses of monomethylated adenine and monomethylated guanine were detected in the MS/MS spectrum.
650.0877	2	1302.1910	1302.1931	-1.6	32.39	mt $tRNA^{Asn}$	11-14	CCAGp	A	2,3,4	1,2,3	1,2,3	1,2,3	
974.1002	1	975.1080	975.1086	-0.6	29.59	mt $tRNA^{Asn}$	15-17	UUGp	M	2,3	1,2	1,2	1,2	
976.1157	1	977.1235	977.1243	-0.7	25.68	mt $tRNA^{Asn}$	15-17	UDGp	M	3	2	1,2	1,2	
815.5976	2	1633.2109	1633.2136	-1.7	36.75	mt $tRNA^{Asn}$	18-22	AUUAGp	M	5	2,3	2	1,2,4	
816.6067	2	1635.2290	1635.2293	-0.2	35.05	mt $tRNA^{Asn}$	18-22	ADUAGp	A	3,4,5	1,2,3,4	1,3	1,2,3,4	
668.0740	1	669.0819	669.0833	-2.1	25.11	mt $tRNA^{Asn}$	25-26,50-51	UGp	M	2	1		1	
1143.1447	2	2288.3050	2288.3065	-0.7	35.63	mt $tRNA^{Asn}$	25-31	U(mmG)CUUAGp	A	2,3,5,7	1,2,3,4,5,6	4,5,6	1,2,3,4,5,6	Bsae loss of dimethylated guanine was detected in the MS/MS spectrum.
803.5927	2	1609.2011	1609.2024	-0.8	33.08	mt $tRNA^{Asn}$	27-31	CUUAGp	A	2,3,4,5	1,2,3,4	1,2,3	1,2,3,4	
803.5931	2	1609.2018	1609.2024	-0.4	34.67	mt $tRNA^{Asn}$	27-31	CUUAGp	A	3,4,5	1,2,3,4	1,2,3	1,2,3,4	
973.1152	1	974.1231	974.1246	-1.6	26.44	mt $tRNA^{Asn}$	32-34	CUGp	M	2,3	1,2	1,2	1,2	
1522.7011	2	3047.4178	3047.4228	-1.7	36.92	mt $tRNA^{Asn}$	35-43	UU(t^6A)ACUAAGp	M	5	2,3,4,5,8	1,2,4,5,	1,2,3,4,5,6,8	Bsae loss of N^6-threonylcarbamoyladenine was detected in the MS/MS spectrum.

1375.5237	3	4129.5945	4129.6049	-2.5	39.53	mt $tRNA^{Asn}$	32-43	CUQUU(t^6A)ACUAAGp	mz					
639.5582	2	1281.1320	1281.1339	-1.5	32.15	mt $tRNA^{Asn}$	46-49	UUUGp	A	2,4	1,2,3	1,2,3	1,2,3	
975.6185	2	1953.2526	1953.2546	-1.0	36.66	mt $tRNA^{Asn}$	54-59	(mU)UUAAGp	A	2,5,6	2,3,4,5	1,2,4	1,2,3,4,5	
1261.6458	2	2525.3072	2525.3103	-1.2	33.65	mt $tRNA^{Asn}$	60-67	UCCCAUUGp	A	2,3,8	2,3,4,5,6,7	4,5,6	2,3,4,5,6,7	
1261.6480	2	2525.3116	2525.3103	0.5	34.28	mt $tRNA^{Asn}$	60-67	UCCCAUUGp	A	2,3,8	2,3,4,5,6,7	3,4,5,6,7	2,3,4,5,6,7	
803.5928	2	1609.2012	1609.2024	-0.8	36.17	mt $tRNA^{Asn}$	69-73	UCUAGp	A	2,4,5	1,2,3,4	1,3	1,2,3,4	
810.6010	2	1623.2176	1623.2181	-0.3	40.61	mt $tRNA^{Asn}$	69-73	UCU(mA)Gp	A	2,3,4,5	1,2,3,4	1,2,3	1,2,3,4	Bsae loss of monomethylated adenine was detected in the MS/MS spectrum.
876.1701	1	877.1779	877.1793	-1.6	32.39	mt $tRNA^{Asn}$	74-76	CCA-OH	M	2	1,2	1,2	2	
1077.0924	1	1078.1002	1078.1022	-1.8	26.06	mt $tRNA^{Gln}$	1-3	pUAGp	M	2	1,2	1,2	2,3	
997.1281	1	998.1359	998.1358	0.1	34.44	mt $tRNA^{Gln}$	5-7,48-50	AUGp	M	3	1,2	1,2	1,2	
721.1120	1	722.1198	722.1211	-1.7	33.14	mt $tRNA^{Gln}$	9-10	(mG)Gp	M	2	1		1	Bsae loss of monomethylated guanine was detected in the MS/MS spectrum.
668.0740	1	669.0819	669.0833	-2.1	25.11	mt $tRNA^{Gln}$	11-12,13-14,20-21	UGp	M	2	1		1	
662.5858	2	1327.1872	1327.1883	-0.9	36.17	mt $tRNA^{Gln}$	15-18	AUAGp	A	2,3,4	1,2,3	1,2	1,2,3	
663.5932	2	1329.2021	1329.2040	-1.5	34.04	mt $tRNA^{Gln}$	15-18	ADAGp	A	2,3,4	1,2,3	1,2,3	1,2,3	
670.0901	1	671.0979	671.0990	-1.6	12.56	mt $tRNA^{Gln}$	20-21	DGp	mz					
650.0889	2	1302.1934	1302.1931	0.2	34.28	mt $tRNA^{Gln}$	23-26	CACGp	A	2,3,4	1,2,3	1,2,3	1,2,3	
691.1022	1	692.1100	692.1105	-0.8	34.75	mt $tRNA^{Gln}$	28-29	AGp	M	2	1	1	1	
1121.6226	2	2245.2608	2245.2643	-1.6	37.79	mt $tRNA^{Gln}$	30-36	AAUUUUGp	mz					
1129.6123	2	2261.2403	2261.2414	-0.5	38.95	mt $tRNA^{Gln}$	30-36	AAUU(s^2U)UGp	M	4,5,7	2,3,4,5,6	1,2	1,2,3,4,5,6	
1198.1191	2	2398.2539	2398.2561	-0.9	39.13	mt $tRNA^{Gln}$	30-36	AAUU(tm^5s^2U)UGp	M	6,7	2,3,4,5,6	1,2,6	1,2,3,4,5,6	
1453.1847	2	2908.3850	2908.3846	0.1	37.36	mt $tRNA^{Gln}$	37-45	(mG)AUUCUCAGp	A	5,9	2,3,4,5,6,7,8	2,3,4,7,8	2,3,4,5,7,8	Bsae loss of monomethylated guanine was detected in the MS/MS spectrum.
1273.6522	2	2549.3201	2549.3215	-0.6	35.63	mt $tRNA^{Gln}$	38-45	AUUCUCAGp	A	4,5,6,8	2,3,4,5,6,7	2,4,7	2,3,4,5,6,7	
639.0659	2	1280.1474	1280.1499	-2.0	29.45	mt $tRNA^{Gln}$	53-56	UUCGp	A	2,3,4	1,2,3	1,2	1,2,3	
646.0748	2	1294.1653	1294.1655	-0.2	32.86	mt $tRNA^{Gln}$	53-56	(mU)UCGp	A	2,4	1,2,3	1,2	1,2,3	
1591.1893	2	3184.3943	3184.3994	-1.6	37.88	mt $tRNA^{Gln}$	57-66	AUUCUCAUAGp	A	3,4,6,10	2,3,5,6,7,8,9	1,3,4,6	1,2,3,4,5,6,7,8,9	
1065.1306	3	3198.4153	3198.4150	0.1	35.95	mt $tRNA^{Gln}$	57-66	(mA)UUCUCAUAGp	A	6,10	3,4,5,6,7,9	1,9	1,2,3,4,5,6,7,8,9	Bsae loss of monomethylated adenine was detected in the MS/MS spectrum.
956.1144	2	1914.2445	1914.2437	0.4	35.54	mt $tRNA^{Gln}$	67-72	UCCUAGp	M	2,3,4,6	2,3,4,5	1,3,4	1,2,3,4,5	
876.1701	1	877.1779	877.1793	-1.6	32.39	mt $tRNA^{Gln}$	73-75	CCA-OH	M	2	1,2	1,2	2	

Supplementary Table 2

stealth siRNA

**Primer Name**	**Sequence (5'-/-3')**	**target gene**
scRNA-for	UGGACGGCUAAAGAAAGCUUGUUAA	TDP-43
scRNA-rev	UUAACAAGCUUUCUUUAGCCGUCCA	TDP-43
siRNA1-for	UUAAGAUCUUUCUUGACCUGCACCA	TDP-43
siRNA1-rev	UGGUGCAGGUCAAGAAAGAUCUUAA	TDP-43
siRNA3-for	GACAGAUGCUUCAUCAGCAGUGAAA	TDP-43
siRNA3-rev	UUUCACUGCUGAUGAAGCAUCUGUC	TDP-43
scRNA-for	CAUGGUUUGAGAGUGUUCCUGGAAA	MRPP3
scRNA-rev	UUUCCAGGAACACUCUCAAACCAUG	MRPP3
scRNA-For	UAGCUUUCAUCUGUAAGCUUCACUU	ELAC2
scRNA-Rev	AAGUGAAGCUUACAGAUGAAAGCUA	ELAC2

Probes for Northen blot analysis

**Primer Name**	**Sequence (5'-/-3')**	**target gene**
COX I	ATTAGGGGAACTAGTCAGTTGCCAAAGCCT	COX I
COX II	TTGACCTCGTCTGTTATGTAAAGGATGCGT	COX II
ND1	TTCATAGTAGAAGAGCGATGGTGAGAGCTA	ND1
ND2	TCCGGAGAGTATATTGTTGAAGAGGATAGC	ND2
CYB	GTTGTGAAGTATAGTACGGATGCTACTTGT	CYB
mirror N	GTGGGTTTAAGTCCCATTGGTCTAG	mirror mt-$tRNA^{Asn}$
mirror A	GCTTAATTAAAGTGGCTGATTTGCGTTCAG	mirror mt-$tRNA^{Ala}$
mirror Q	GGATTCTCAGGGATGGGTTCGATTCTCATA	mirror mt-$tRNA^{Gln}$
N	CTAGACCAATGGGACTTAAACCCACAAACACTTA	mt-$tRNA^{Asn}$
Q	TATGAGAATCGAACCCATCCCTGAGAATCC	mt-$tRNA^{Gln}$
mt-$tRNA^{Pro}$	GTCTTTAACTCCACCATTAGCACCCAAAGC	mt-$tRNA^{Pro}$
mt-$tRNA^{Cys}$	AAGCCCCGGCAGGTTTGAAGCTGCTTCTTC	mt-$tRNA^{Cys}$
mt-$tRNA^{Ala}$	CTGAACGCAAATCAGCCACTTTAATTAAGC	mt-$tRNA^{Ala}$
mt-$tRNA^{Ser(UCN)}$	CATGAGTTAGCAGTTCTTGTGAGCTTTCTC	mt-$tRNA^{Ser(UCN)}$
mt-$tRNA^{Tyr}$	CAGTCCAATGCTTCACTCAGCCATTTTACC	mt-$tRNA^{Tyr}$
mt-$tRNA^{Glu}$	GCACGGACTACAACCACGACCAATGATATG	mt-$tRNA^{Glu}$
L	TATGCGATTACCGGGCTCTGCCATCTTAAC	mt-$tRNA^{Leu(UUR)}$
mt-$tRNA^{Trp}$	CAGAAATTAAGTATTGCAACTTACTGAGGGC	mt-$tRNA^{Trp}$
mt-$tRNA^{His}$	AATCTGATGTTTTGGTTAAACTATATTTAC	mt-$tRNA^{His}$
mt-$tRNA^{Lys}$	TTTAACTTAAAAGGTTAATGCTAAGTTAGC	mt-$tRNA^{Lys}$
mt-$tRNA^{Gly}$	AAGTTAACGGTACTATTTATACTAAAAGAG	mt-$tRNA^{Gly}$
mt-$tRNA^{Phe}$	CAGTGTATTGCTTTGAGGAGGTAAGCTACA	mt-$tRNA^{Phe}$
mt-$tRNA^{Val}$	GTTGGGTGCTTTGTGTTAAGCTACACTCTG	mt-$tRNA^{Val}$
16S rRNA	GGGTAAATGGTTTGGCTAAGGTTGTCTGGT	16S rRNA
U1 snRNA	ATACTTACCTGGCAGGGGAGATAC	U1 snRNA
5.8S rRNA	AGACAGGCGTAGCCCCGGGAGGAA	5.8S rRNA
$tRNA^{Met}$	TAGCAGAGGATGGTTTCGATCCATCGA	$tRNA^{Met}$

Primers for qPCR

**Primer Name**	**Sequence (5'-/-3')**	**target gene**
COX I-for	CTGCTATAGTGGAGGCCGGA	COX I
COX I-rev	GGGTGGGAGTAGTTCCCTGC	COX I
COX II-for	TGCCCGCCATCATCCTA	COX II
COX II-rev	TCTGTTATGTAAAGGATGCGT	COX II
COX III-for	CCAATGATGGCGCGATG	COX III
COX III-rev	CTTTTTGGACAGGTGGTGTGTG	COX III
CYB-for	ACATCGGCATTATCCTCCTG	CYB
CYB-rev	GTGTGAGGGTGGGACTGTCT	CYB
ND1-for	CATATGACGCACTCTCCCCT	ND1
ND1-rev	GGGGAATGCTGGAGATTGTA	ND1
ND2-for	AAGCAACCGCATCCATAATC	ND2
ND2-rev	TCAGAAGTGAAAGGGGGCTA	ND2
ND3-for	ACCACAACTCAACGGCTACA	ND3
ND3-rev	TTGTAGGGCTCATGGTAGGG	ND3
ND4-for	CCATTCTCCTCCTATCCCTCAAC	ND4
ND4-rev	CACAATCTGATGTTTTGGTTAAACTATATTT	ND4
ND4L-for	TCGCTCACACCTCATATCCTC	ND4L
ND4L-rev	GGCCATATGTGTTGGAGATTG	ND4L
ND5-for	AGTTACAATCGGCATCAACCAA	ND5
ND5-rev	CCCGGAGCACATAAATAGTATGG	ND5
ND6-for	ATCCTACCTCCATCGCTAAC	ND6
ND6-rev	AGCCTTCTCCTATTTATGGG	ND6
ATP6-for	GCGGGCACAGTGATTATAGG	ATP6
ATP6-rev	AGGTGGCCTGCAGTAATGTT	ATP6
ATP8-for	TACTACCGTATGGCCCACCA	ATP8

ATP8-rev	TTTGGTGAGGGAGGTAGGTG	ATP8
16S rRNA-for	TTTACGACCTCGATGTTGGATCA	16S rRNA
16S rRNA-rev	CGGTCTGAACTCAGATCACGTA	16S rRNA
ACTB-for	CATGTACGTTGCTATCCAGGC	ACTB
ACTB-rev	CTCCTTAATGTCACGCACGAT	ACTB
TDP-43 3'UTR-for	GCATGTTCAAAACGGAAACCATTG	TDP-43 coding region
TDP-43 3'UTR-rev	AACACTGTGACACCATGATCTCC	TDP-43 coding region
TDP-43 code-for	GTGTGGGCTTCGCTACAGG	TDP-43 3'UTR
TDP-43 code-rev	CAACATACACCAGATTTCCCCAG	TDP-43 3'UTR
GAPDH-for	CATGAGAAGTATGACAACAGCCT	GAPDH
GAPDH-rev	AGTCCTTCCACGATACCAAAGT	GAPDH
ND6-RT	CCTATTCCCCCGAGCAATCTCAATTACAAT	ND6

Primers for sqRT-PCR

**Primer Name**	**Sequence (5'-/-3')**	**target gene**
Alaf	CTGAACGCAAATCAGCCACTTTAATTAAGC	Ala-Tyr
Tyrr	GGTAAAATGGCTGAGTGAAGCATTGGACTG	Ala-Tyr
Glnf	TATGAGAATCGAACCCATCCCTGAGAATCC	Gln-ND2
mND2r	TCAGAAGTGAAAGGGGGCTA	Gln-ND2
Tyrf	CAGTCCAATGCTTCACTCAGCCATTTTACC	Tyr-COXI
mCOXIr	TAGGGGAACTAGTCAGTTGCCAAAG	Tyr-COXI
Gluf	GCACGGACTACAACCACGACCAATGATATG	Glu-CYB
mCYBr	AGAATATTGAGGCGCCATTG	Glu-CYB
Ala-RT	CTGAACGCAAATCAGCCACTTTAATTAAGC	
Gln-RT	TATGAGAATCGAACCCATCCCTGAGAATCC	
Tyr-RT	CAGTCCAATGCTTCACTCAGCCATTTTACC	
Glu-RT	GCACGGACTACAACCACGACCAATGATATG	
GAPDH-for	CATGAGAAGTATGACAACAGCCT	GAPDH
GAPDH-rev	AGTCCTTCCACGATACCAAAGT	GAPDH
GAPDH-RT	AGTCCTTCCACGATACCAAAGT	
ND2-for	AAGCAACCGCATCCATAATC	ND2-mirrorN
mirrorN-rev	GTGGGTTTAAGTCCCATTGGTCTAG	ND2-mirrorN
ND1-for	CATATGACGCACTCTCCCCT	ND1-mirrorN
ND1-rev	GTGGGTTTAAGTCCCATTGGTCTAG	ND1-mirrorN
mirrorAla-for	CTGAACGCAAATCAGCCACTTTAATTAAGC	mirrorA-COX I
COX I-rev3	AGGGTAGACTGTTCAACCTG	mirrorA-COX I
5S-for	TACGGCCATACCACCCTGAA	5S rRNA
5S-rev	GCGGTCTCCCATCCAAGTAC	5S rRNA
ACTB-for	CATGTACGTTGCTATCCAGGC	ACTB
ACTB-rev	CTCCTTAATGTCACGCACGAT	ACTB

Construct

**Primer Name**	**Sequence (5'-/-3')**	**target gene**
KpnI-FL-TDP43-1-For	TATATAGGTACCGCCACCATGGACTACAAGGACGACGACGACAAGGG ATCCATGTCTGAATATATTCGGGTA	FLAG-TDP-43
TDP43(-414)-XhoI-Rev	TATATACTCG AGCTACATTC CCCAGCCAGA AGA	FLAG-TDP-43
HindIII-6xHis-BT-For	TATATAAGCTTGCCACCATGCACCACCACCACCAC	6xHis-BT
6xHis-BT-KpnI-Rev	TATAGGTACCCGCCAGGGTCATCAGGGTGTC	6xHis-BT
ClaI-TDP43(170-)-For	CCCATCGATG GACGATGGTG TGACTGCAAA	ΔRRM1
TDP43(-104)-ClaI-Rev	CCCATCGATG GATGTTTTCT GGACTGCTCT	ΔRRM1
ClaI-TDP43(258-)-For	CCCATCGATT CCAATGCCGA ACCTAAGCAC	ΔRRM2
TDP43(-192)-ClaI-Rev	CCCATCGATT TTTCTGCTTC TCAAAGGCTC	ΔRRM2
ClaI-TDP43(315-)-For	TATATCGATG CGTTCAGCAT TAATCCAGCC	ΔGR
TDP43(-273)-ClaI-Rev	CCCATCGATA CTTCTTTCTA ACTGTCTATT	ΔGR
BamHI-TDP43-1-For	TATATAGGAT CCATGTCTGA ATATATTCGG GTA	Δ315
TDP43(-314)-XhoI-Rev	ATACTCGAGC TAACCAAAGT TCATCCCACC ACC	Δ315
K136A-For	TCTTATGGTG CAGGTCGCGA AAGATCTTAA GACTG	K136A
K136A-Rev	CAGTCTTAAGATCTTTCGCGACCTGCACCATAAGA	K136A
K140A-For	GGTCAAGAAA GATCTTGCGA CTGGTCATTC AAAGG	K140A
K140A-Rev	CCTTTGAATGACCAGTCGCAAGATCTTTCTTGACC	K140A
K145A-For	TTAAGACTGG TCATTCAGCG GGGTTTGGCT TTGTT	K145A
K145A-Rev	AACAAAGCCAAACCCCGCTGAATGACCAGTCTTAA	K145A
F147/149L-For	GTCATTCAAA GGGGTTAGGC TTAGTTCGTT TTACGGAATA	F147/149L
F147/149L-Rev	TATTCCGTAA AACGAACTAA GCCTAACCCC TTTGAATGAC	F147/149L
pCold-FLAG-For	GAAGAAAAGCTTGGTGCCGATTACAAGGATGACGACGATAAGTAATCT AGAGAAGAA	pCold-TF-FLAG
pCold-FLAG-Rev	TTCTTCTCTAGATTACTTATCGTCGTCATCCTTGTAATCGGCACCAAGC TTTTCTTC	pCold-TF-FLAG
BamHI-TDP43-HindIII-For	GCGCGGGATCCATGTCTGAATATATTCGGGTAA	pCold-TF-TDP43-FLAG
BamHI-TDP43-HindIII-Rev	CGCGCAAGCTTCATTCCCCAGCCAGAAGACTTAGA	pCold-TF-TDP43-FLAG

In vitro transcription

Primer Name	Sequence (5'-/-3')	target gene
T7-for	CTAATACGAC TCACTATAGG GAGA	T7-mt-tRNA(Asn)
T7-mt-tRNA(Asn)-rev	CTAATACGAC TCACTATAGG GAGATAGATT GAAGCCAGTT<br>GATTAGGGTG CTTAGCTGTTAACTAAGTGT TTGTGGGTTT<br>AAGTCCCATT GGTCTA	T7-mt-tRNA(Asn)
T7-for	CTAATACGAC TCACTATAGG GAGA	T7-AC-stem(Leu)
T7-mt-tRNA(Asn) AC-stem(Leu)-rev	CTAATACGAC TCACTATAGG GAGAGTTAAG AAAGCCAGTT<br>GATTAGGGTG CTTAGCTGTTAACTAAGTGT TTGTGGGTTT<br>AAGTCCCATT CTTAACA	T7-AC-stem(Leu)
T7-for	CTAATACGAC TCACTATAGG GAGA	T7-D-loop(Leu)
T7-mt-tRNA(Asn) D-loop(Leu)-rev	CTAATACGAC TCACTATAGG GAGATAGATT GAAGCAGAGC<br>CCGGTAATCG TGCTTAGCTGTTAACTAAGT GTTTGTGGGT<br>TTAAGTCCCA TTGGTCTAG	T7-D-loop(Leu)
T7-for	CTAATACGAC TCACTATAGG GAGA	T7-pAntiCdn(Leu)
T7-mt-tRNA(Asn) pAntiCdn(Leu)-rev	CTAATACGAC TCACTATAGG GAGATAGATT GAAGCCAGTT<br>GATTAGGGTG CTTAGCTTAAAACTAAGTGT TTGTGGGTTT<br>AAGTCCCATT GGTCTAG	T7-pAntiCdn(Leu)
T7-for	CTAATACGAC TCACTATAGG GAGA	T7-Var-R(Leu)
T7-mt-tRNA(Asn) Var-R(Leu)-rev	CTAATACGAC TCACTATAGG GAGATAGATT GAAGCCAGTT<br>GATTAGGGTG CTTAGCTGTT AACTAAGCAG TCGTGGGTTT<br>AAGTCCCATT GGTCTAG	T7-Var-R(Leu)
T7-for	CTAATACGAC TCACTATAGG GAGA	T7-T-loop(Leu)
T7-mt-tRNA(Asn) T-loop(Leu)-rev	CTAATACGAC TCACTATAGG GAGATAGATT GAAGCCAGTT<br>GATTAGGGTG CTTAGCTGTT AACTAAGTGT TTGGAGGTTC<br>AATTCCTCTT GGTCTAG	T7-T-loop(Leu)
T7-for	CTAATACGAC TCACTATAGG GAGA	T7-Leu(UUR)
T7-mt-tRNA(Leu-UUR)-rev	CTAATACGAC TCACTATAGG GAGAGTTAAG ATGGCAGAGC<br>CCGGTAATCG CATAAAACTTAAAACTTTAC AGTCAGAGGT<br>TCAATTCCTC TTCTTAACA	T7-Leu(UUR)
mirrorANCY-COXI-for	CGCGGATCCTAAGGACTGCAAAACCCCACTCTGC	mirrorANCY-COXI pSPT19
mirrorANCY-COXI-rev	TCTAGATTTTATGTATACGGG	mirrorANCY-COXI pSPT19

Supplementary Figure 1

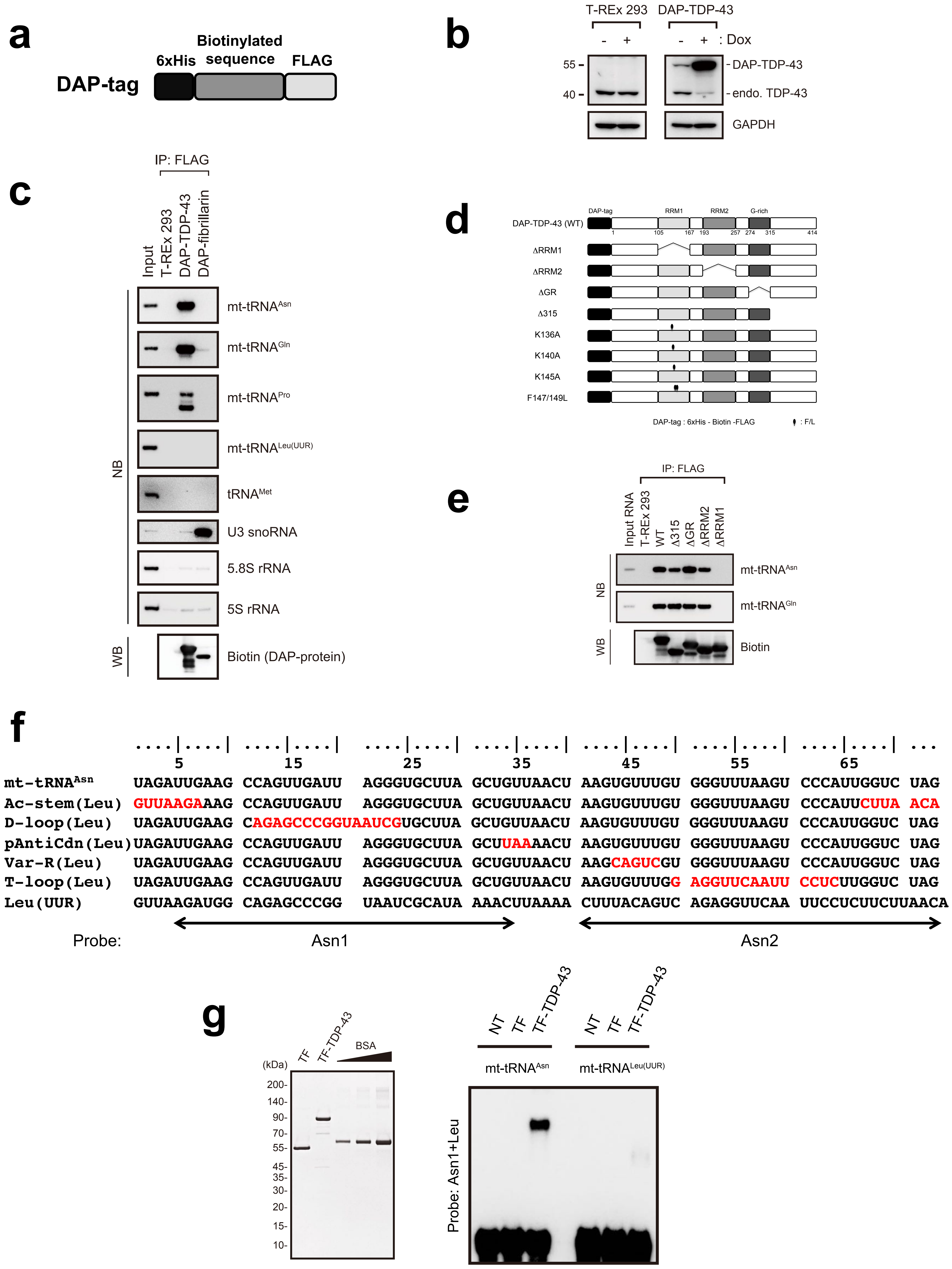

Supplementary Figure 2

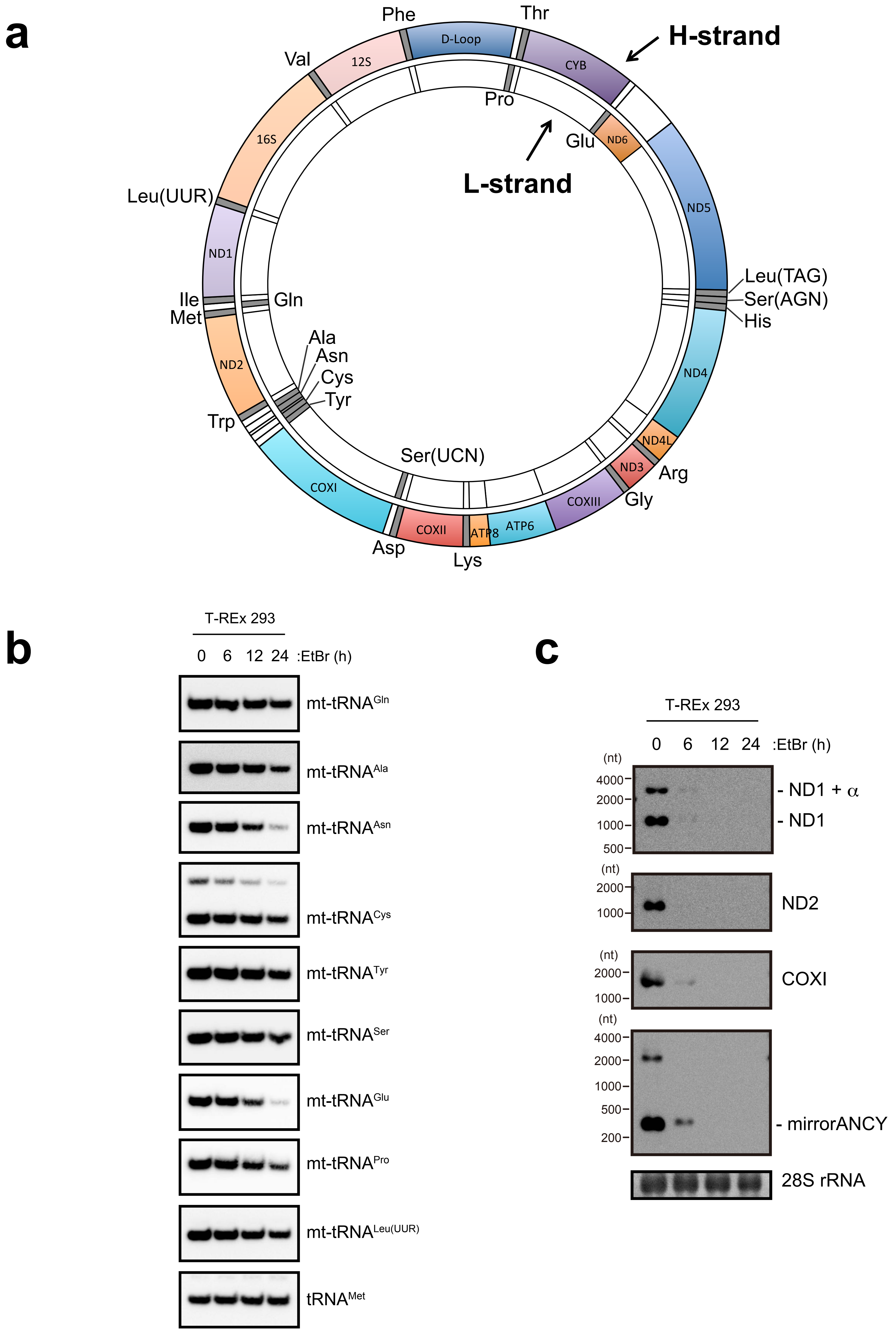

Supplementary Figure 3

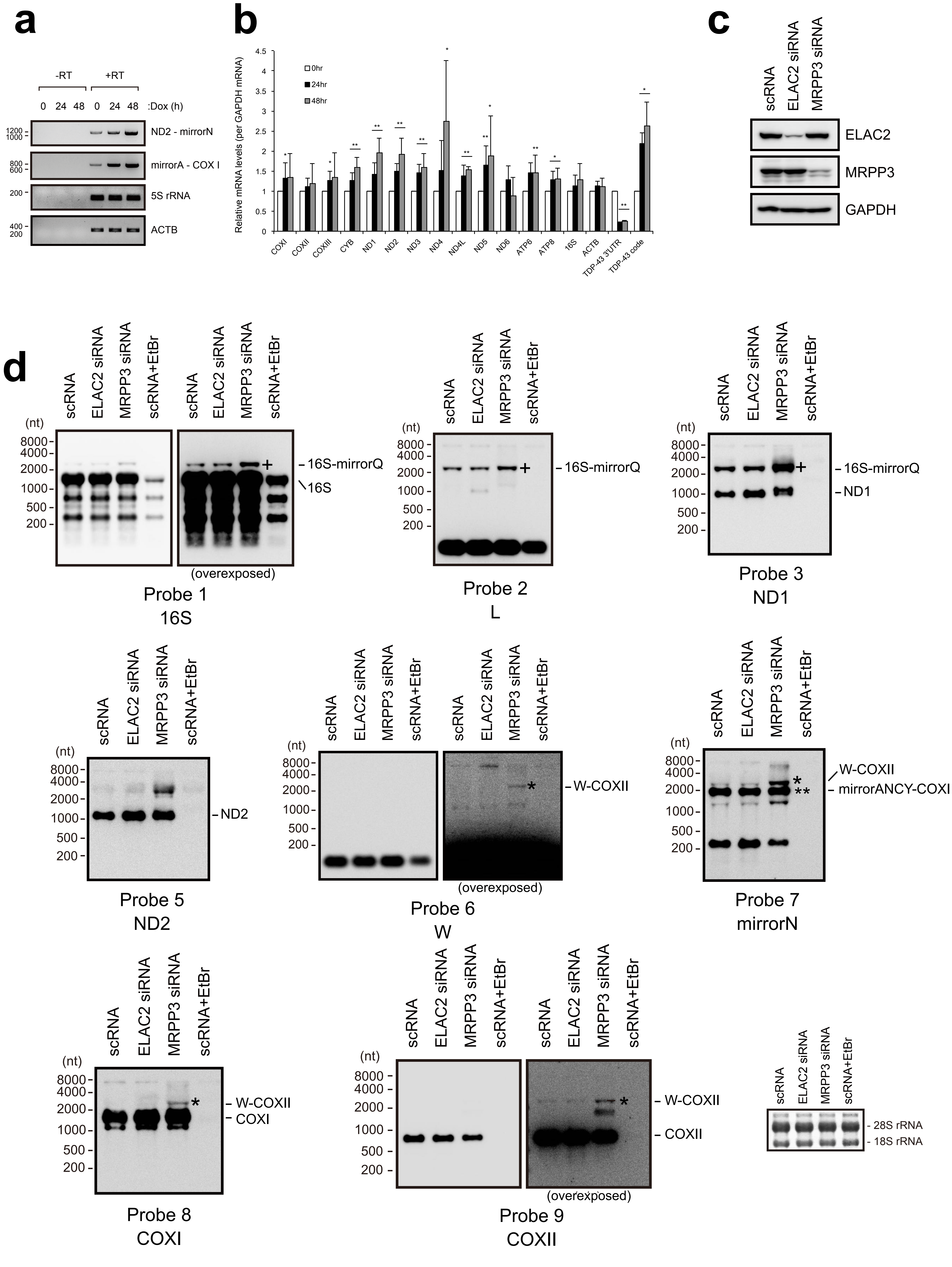

Uncropped images-1

Figure 1a

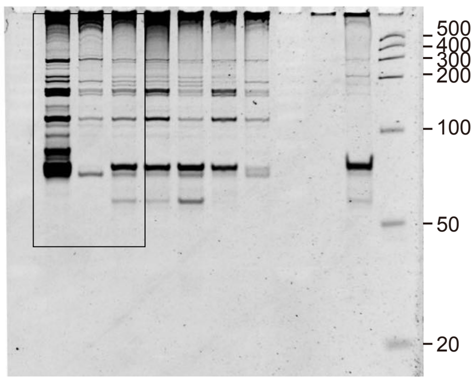

Figure 1c

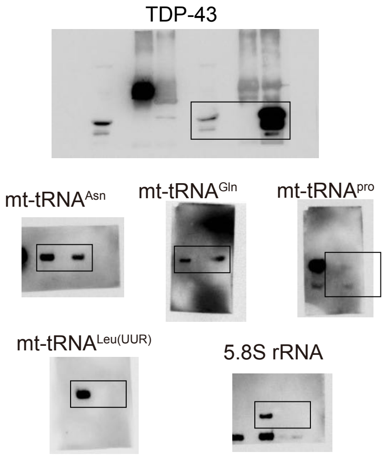

Figure 1d

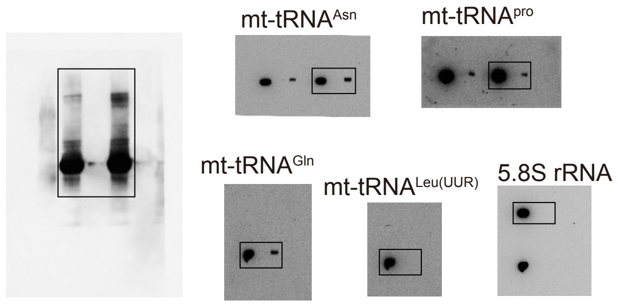

Figure 1e

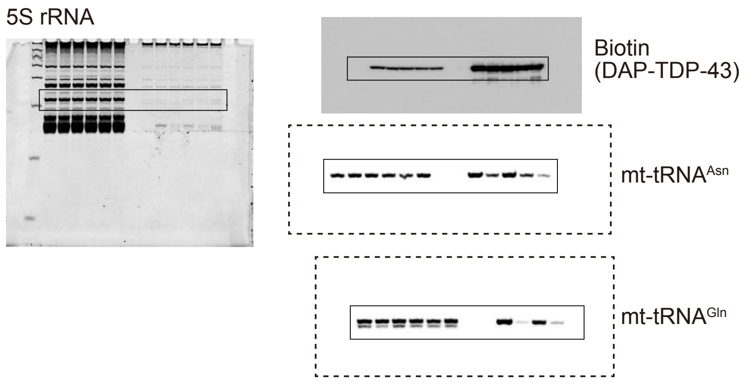

Figure 1f

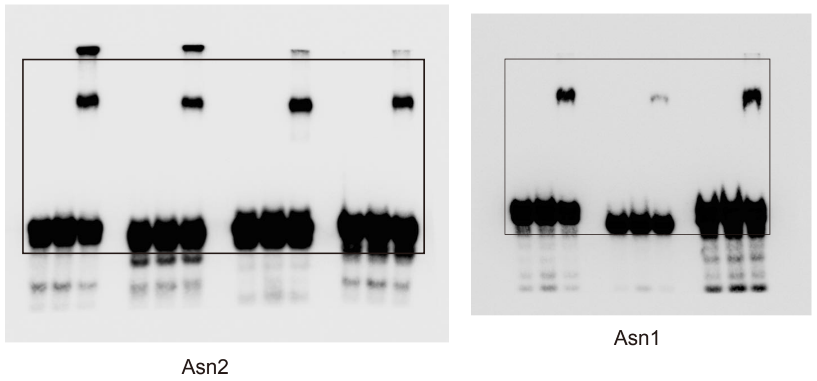

Figure 1g

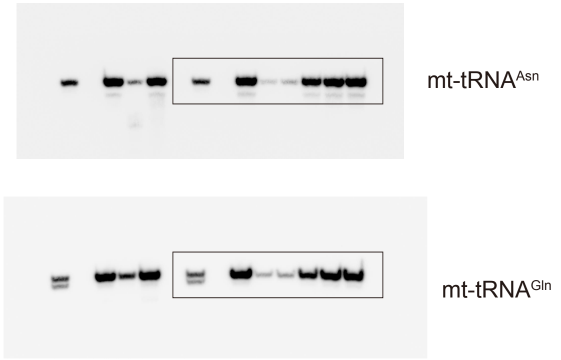

Figure 2a

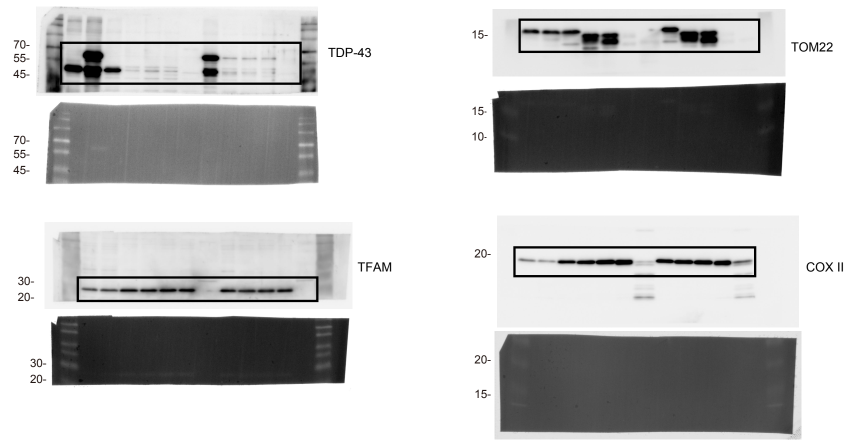

Figure 3a

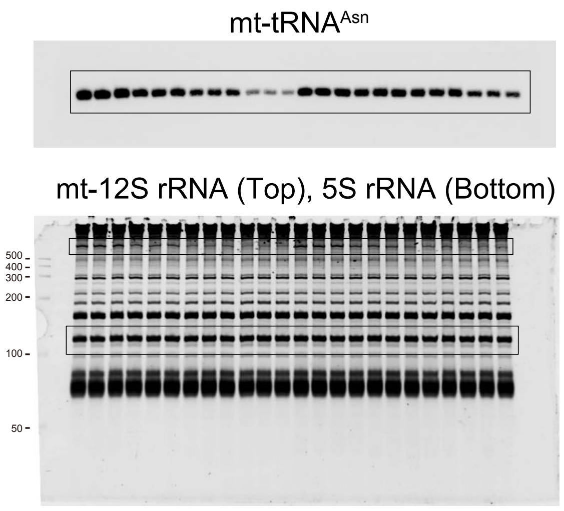

Figure 3b

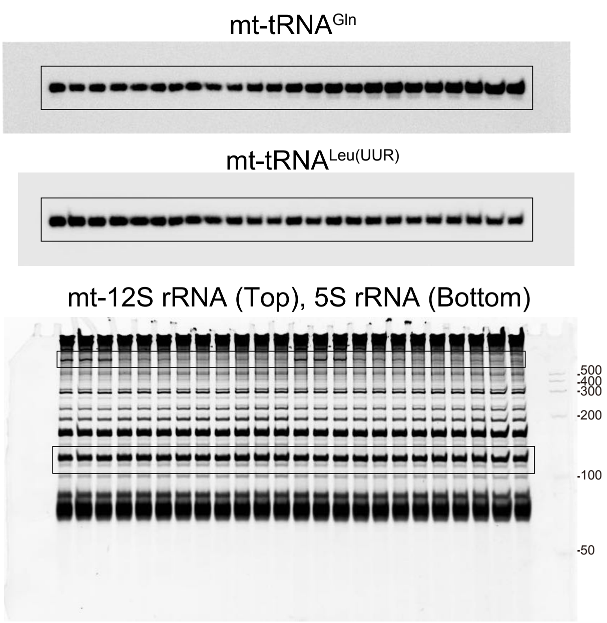

Uncropped images-2

Figure 4b

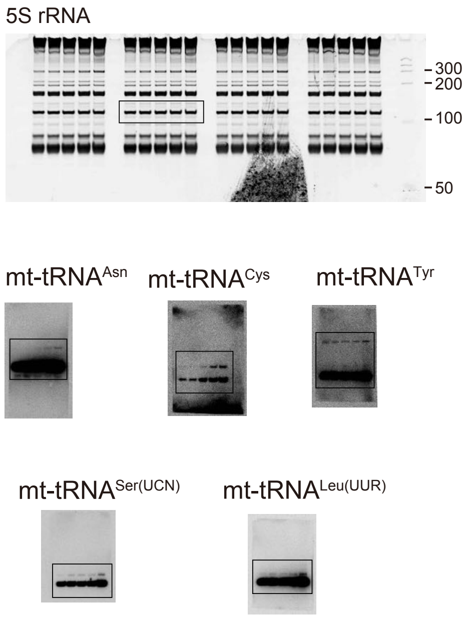

Figure 4c

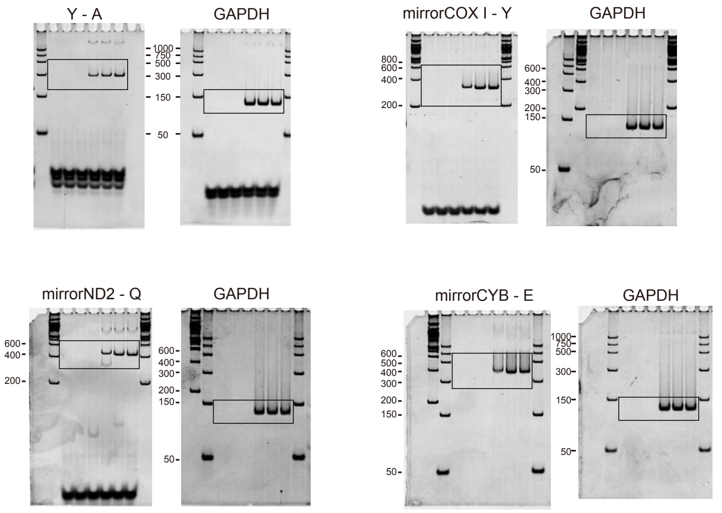

Figure 4d

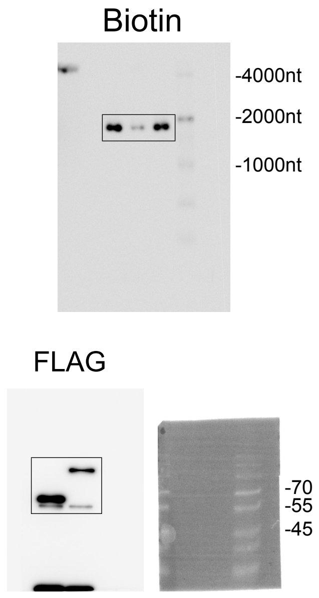

Figure 6d

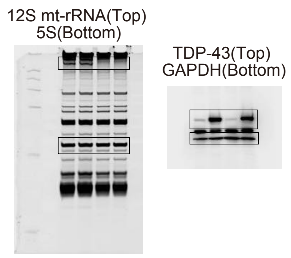

Figure 5b

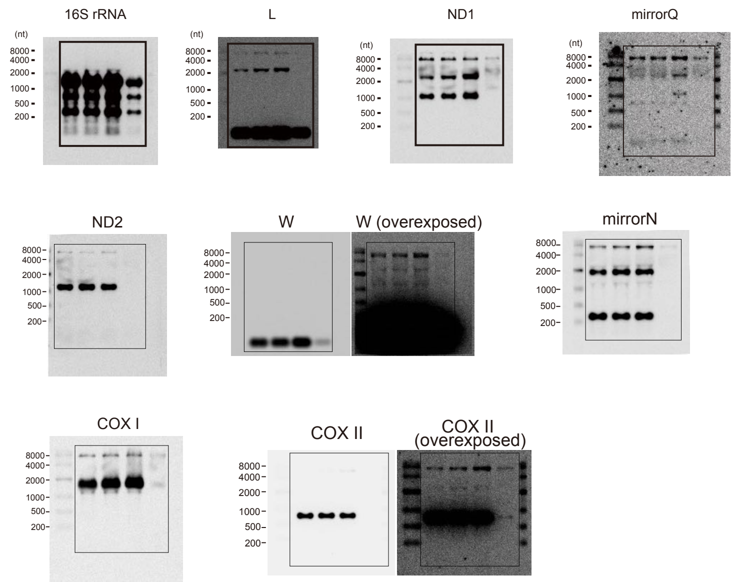

Figure 4e

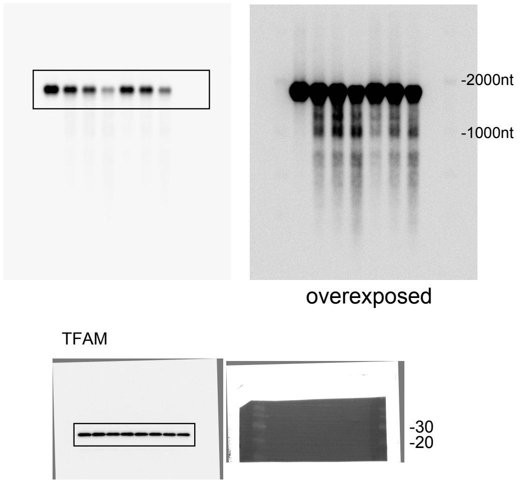

Figure 6e

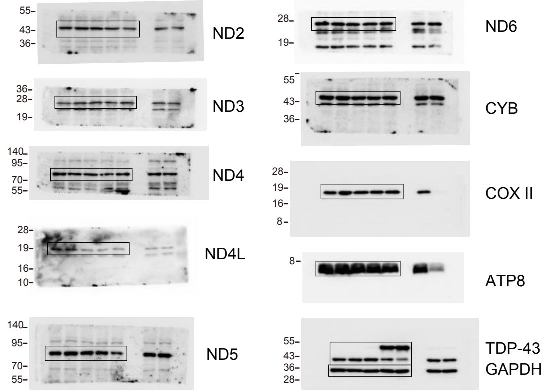

Figure 7d

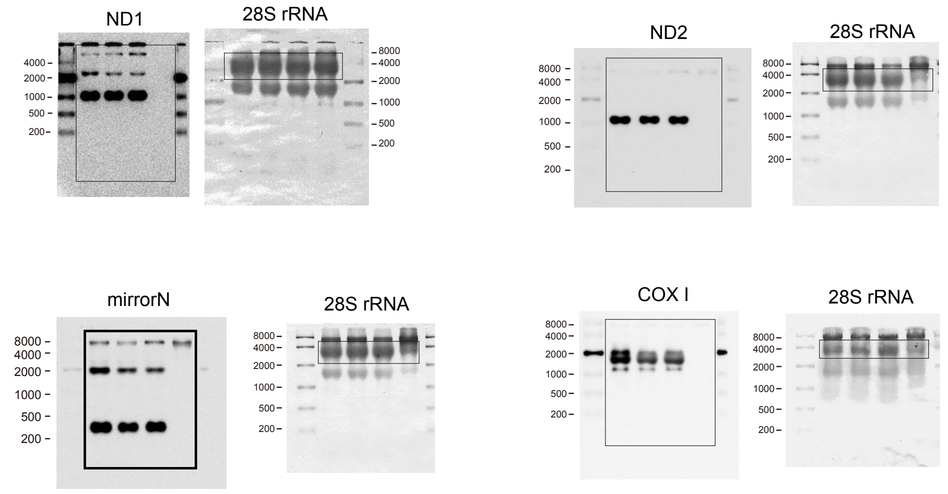

Figure 7a

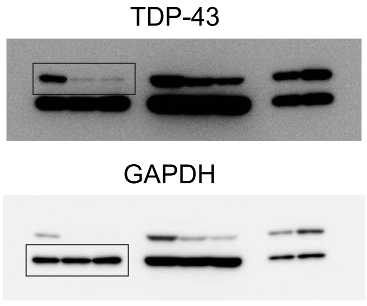

Uncropped images-3

Sup. Figure 1b

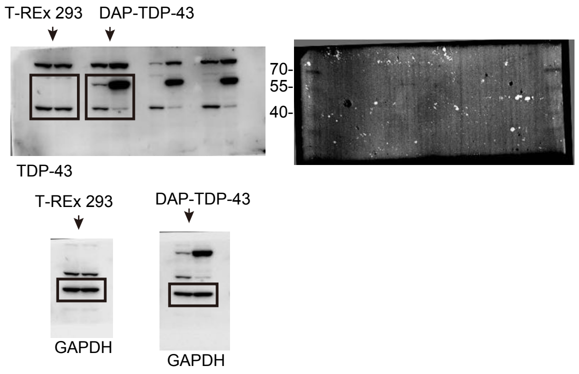

Sup. Figure 1c

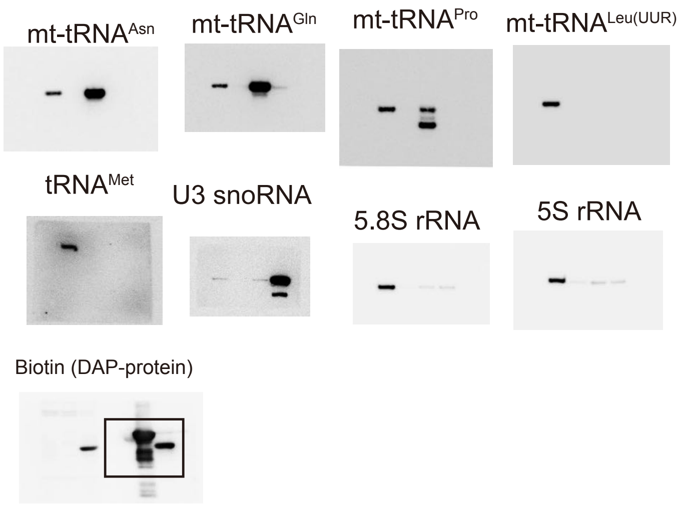

Sup. Figure 1e

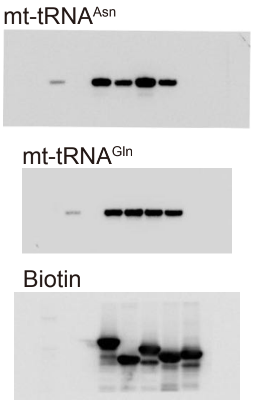

Sup. Figure 1g

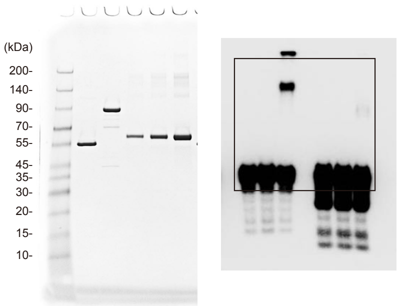

Sup. Figure 2b

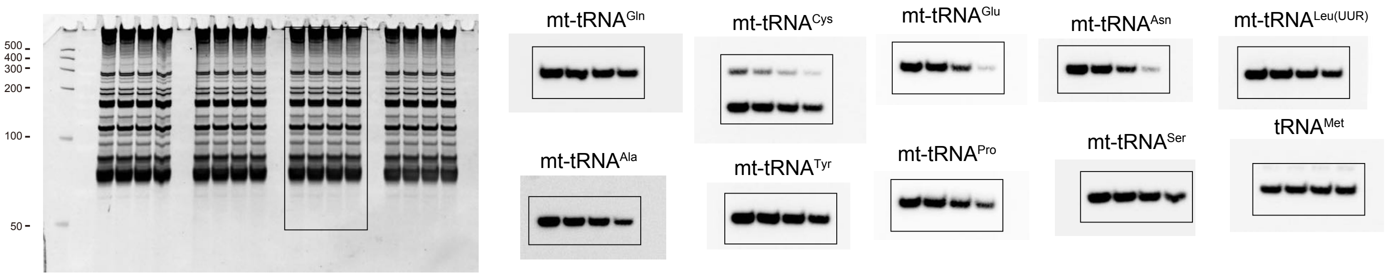

Sup. Figure 2c

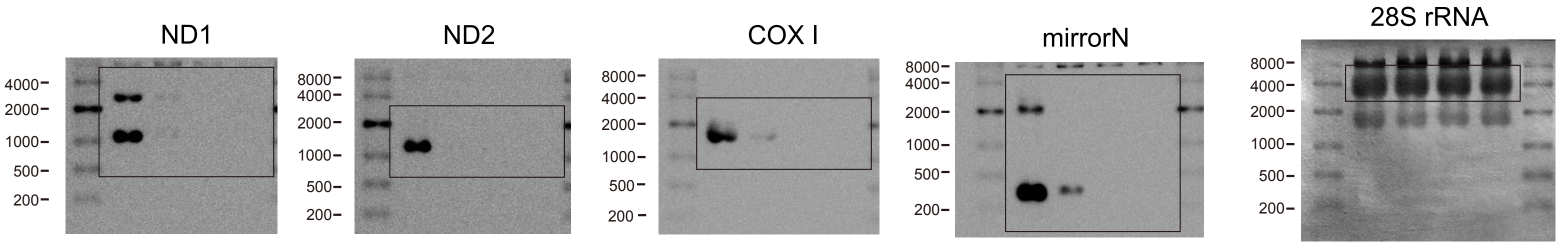

Sup. Figure 3a

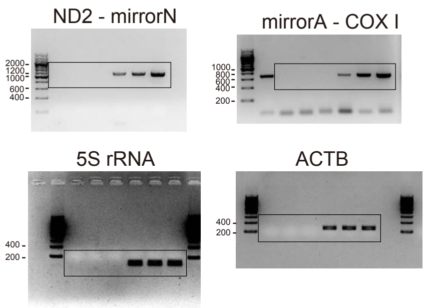

Sup. Figure 3d

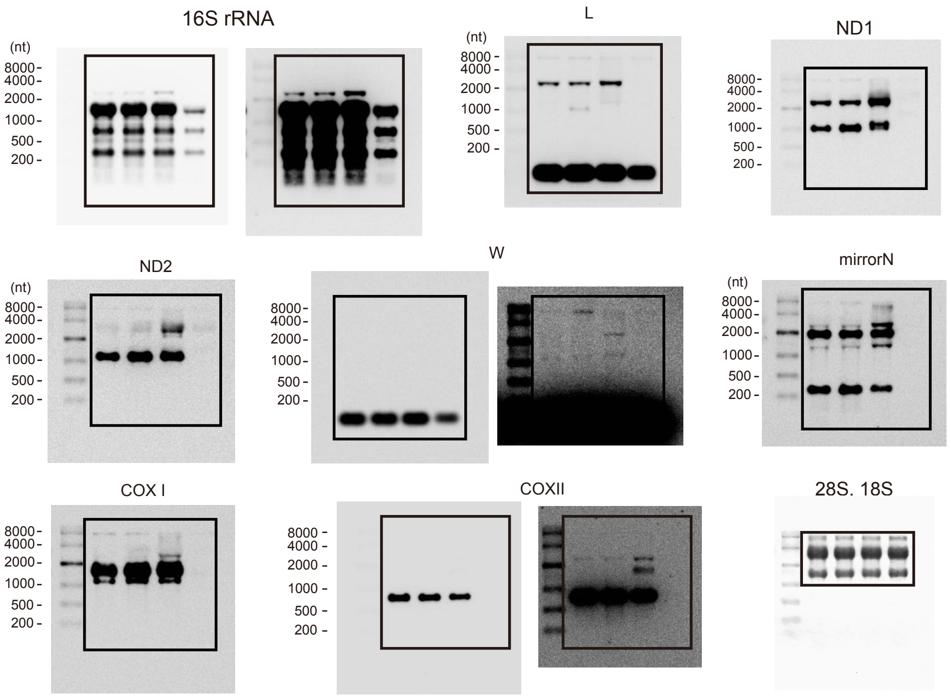

Sup. Figure 3c

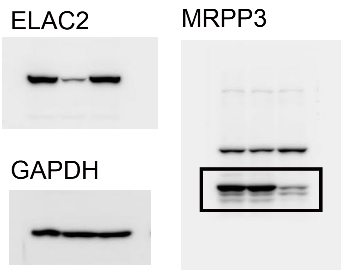

Supplement: Supplementary file 1 — Supplementary Information [file 41598_2017_6953_MOESM1_ESM.pdf]
